# Complete Chloroplast Genome Sequences of Important Oilseed Crop *Sesamum indicum* L

**DOI:** 10.1371/journal.pone.0035872

**Published:** 2012-05-14

**Authors:** Dong-Keun Yi, Ki-Joong Kim

**Affiliations:** School of Life Sciences, Korea University, Seoul, Korea; University of Arizona, United States of America

## Abstract

*Sesamum indicum* is an important crop plant species for yielding oil. The complete chloroplast (cp) genome of *S. indicum* (GenBank acc no. JN637766) is 153,324 bp in length, and has a pair of inverted repeat (IR) regions consisting of 25,141 bp each. The lengths of the large single copy (LSC) and the small single copy (SSC) regions are 85,170 bp and 17,872 bp, respectively. Comparative cp DNA sequence analyses of *S. indicum* with other cp genomes reveal that the genome structure, gene order, gene and intron contents, AT contents, codon usage, and transcription units are similar to the typical angiosperm cp genomes. Nucleotide diversity of the IR region between *Sesamum* and three other cp genomes is much lower than that of the LSC and SSC regions in both the coding region and noncoding region. As a summary, the regional constraints strongly affect the sequence evolution of the cp genomes, while the functional constraints weakly affect the sequence evolution of cp genomes. Five short inversions associated with short palindromic sequences that form step-loop structures were observed in the chloroplast genome of *S. indicum*. Twenty-eight different simple sequence repeat loci have been detected in the chloroplast genome of *S. indicum*. Almost all of the SSR loci were composed of A or T, so this may also contribute to the A-T richness of the cp genome of *S. indicum*. Seven large repeated loci in the chloroplast genome of *S. indicum* were also identified and these loci are useful to developing *S. indicum*-specific cp genome vectors. The complete cp DNA sequences of *S. indicum* reported in this paper are prerequisite to modifying this important oilseed crop by cp genetic engineering techniques.

## Introduction

*Sesamum indicum* L. cv. Ansanggae is an annual plant that reaches 50 to 100 cm tall. Commonly known as sesame or til, *S. indicum* is an important and ancient oil-yielding crop. Sesame seeds are cultivated as a rich source of edible oil. Though the origin is uncertain, the species probably originated from southeastern Africa and a naturalized population has been found in India [1], [2]. *S. indicum* is widely cultivated and naturalized in dry habitats of tropical and subtropical regions, with the primary production occurring in the developing countries of Asia and Africa. Sesame seeds contain approximately 50–60% edible oil, and sesame oil is ranked 5^th^ in terms of oil production. Sesame seed production worldwide is estimated at 4 million tons and production is steadily growing. Nearly 70% of the world’s production is consumed in the producing countries and world trade is limited. The major producing countries in descending order are: Myanmar (867,520 tons), India (657,000 tons), China (622,905 tons), Sudan (318,000 tons), Ethiopia (260,534 tons), Uganda (178,000 tons), and Nigeria (110,000 tons). *S. indicum* is a member of the family Pedaliaceae, order Lamiales. This order also includes the family Oleaceae.

In comparison with sunflower, canola, and soybeans, crops which are primarily cultivated in advanced countries, the modern genetic research on sesame has been relatively limited [3], [4]. This is because *S. indicum* is mostly cultivated in developing countries. Most studies have focused on the nutrients and products of sesame. Most recently, ISSR markers and EST tags were developed for the creation of genetic maps in sesame [5], [6], [7]. To date, there have been no known studies of the sesame chloroplast (cp) genome sequence. Therefore, the complete cp genome sequences of *S. indicum* were generated and characterized for their suitability as cp genome vector sequences for application in future genetic engineering studies.

The genomes of chloroplasts, the plant organelles responsible for photosynthesis [8], [9], provide rich evolutionary and phylogenetic information [10], [11]. Accordingly, several recent studies have used cp genome information to construct the angiosperm phylogeny [12], [13]. The complete cp genomes of more than 170 species, including many crop species, have been reported from various groups of plants and algae (Chloroplast Genome Database, http://chloroplast.cbio.psu.edu).

The majority of the cp genomes of land plants contain 90–110 unique genes within the 115–165 kb of circular chromosome [14]. The primary mechanism of gene order change is inversion by intramolecular recombination, and this method occurs principally via the dispersed repeats of the cp genome [15], [16]. Evolutionary hot spots showing high levels of insertions and deletions (indels) with high incidences of base substitutions are concentrated on specific gene and intergenic spacers [17]. Several comparative studies have documented the phylogenetic usefulness of cp genome structures at higher taxonomic levels [18], [19]. However, only a few studies have explored the usefulness of cp genome data in closely related taxa.

Currently, transformation using chloroplast vectors provides a valuable technique for chloroplast genetic engineering [20]. Cp genome vectors show high-levels of gene expression, the possibility of the expression of multiple genes or pathways via a single transformation event, and transgene containment due to a lack of pollen transmission [21]. Gene transformation protocols using universal cp genome vectors have been developed in tobacco and carrots [22], [23]. However, the universal vectors show limited utility for distantly related plant species. To construct a species-specific cp genome vector, the complete cp DNA sequence is necessary. For this purpose, the complete cp DNA sequences from *S. indicum* (Pedaliaceae) are reported herein. In addition, a comparative sequence analysis of the whole cp genomes of *S. indicum* and *O. europaea* was conducted to reveal more information concerning recent cp genome evolution. The comparative data will contribute to an increased understanding of the evolutionary model of the cp genome in the order Lamiales. To develop gene transformation protocols using a chloroplast genome vector, an analysis was performed on the repeating sites within the chloroplast genome. This information may enable the production of sesame-specific chloroplast genome vectors.

## Results

### General Features of the *Sesamum Indicum* cp Genome

**Figure 1:**
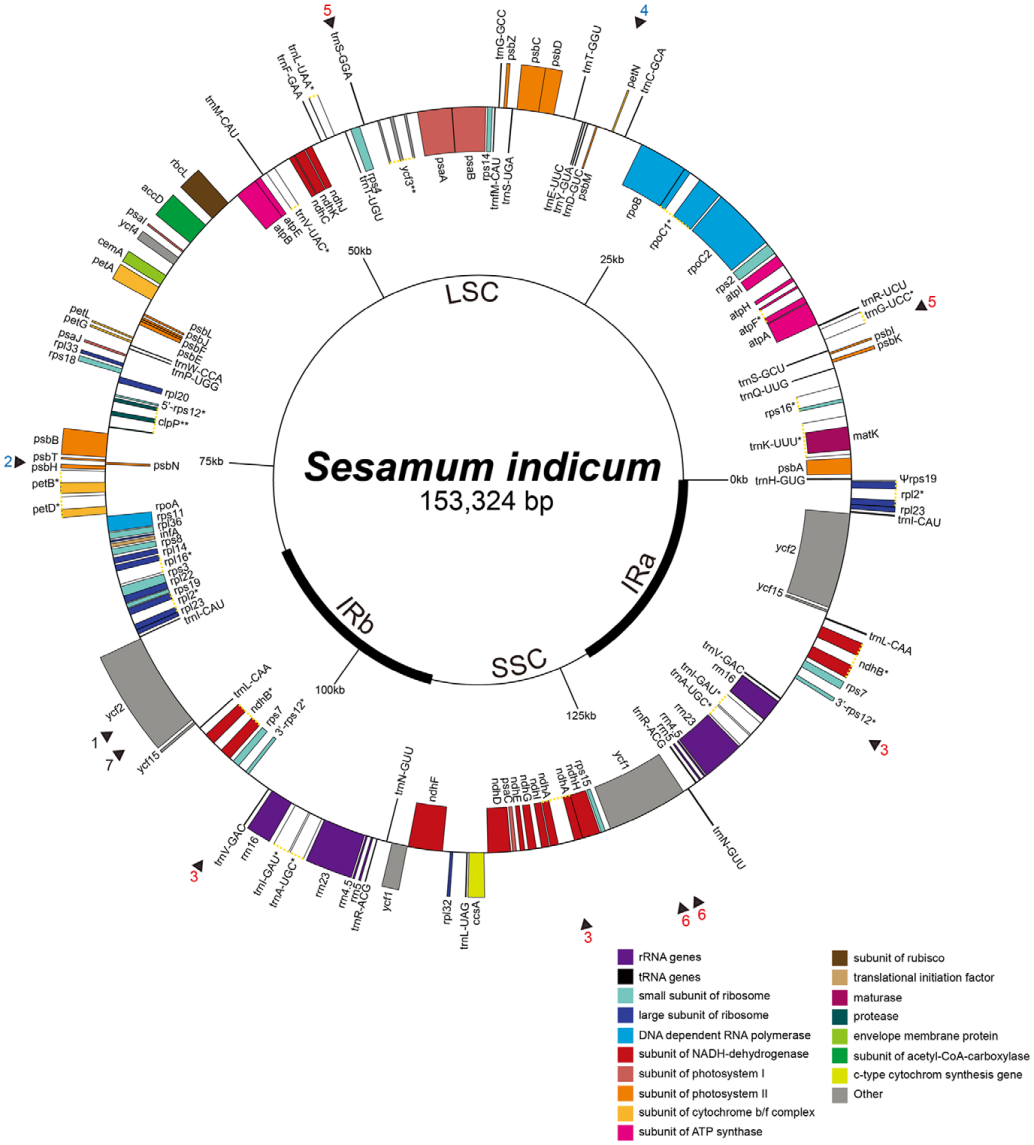
The gene map of *Sesamum indicum* cp genome. A pair of thick lines at the inside circle represents the inverted repeats (IRa and IRb; 25,141 bp each), which separate the large single copy region (LSC; 85,170 bp) from the small single copy region (SSC; 17,872 bp). Genes drawn inside the circle are transcribed clockwise, while those drawn outside the circle are transcribed counterclockwise. Intron-containing genes are marked by asterisks. The numbers at the outmost circle indicate the locations of 7 repeats including direct (black number), palimdromic (blue number), and dispersed repeats (red numbers), respectively (cf. Table 4).

The *Sesamum indicum* cp genome exhibits the general cp genome structure characteristic of flowering plants. It contains a pair of inverted repeat regions (IRa and IRb) that comprise 25,141 bp each. The two IR regions divide the genome into a large single copy (LSC) region and a small single copy (SSC) region. The LSC region is 85,170 bp, whereas the SSC region is 17,872 bp. The complete cp sequence of *S. indicum* is 153,324 bp in length (GenBank acc no. JN637766), of which 58% is coding regions and 42% is non-coding regions. A total of 114 genes are contained within the *S. indicum* cp genome, including 80 protein-coding genes, 30 transfer RNA genes, and four ribosomal RNA genes (Figure 1, Table 1). Ten protein-coding and seven tRNA coding genes are duplicated on the IR regions. The LSC region contains 62 protein-coding and 22 tRNA genes, while the SSC region contains 12 protein-coding and one tRNA gene. Similar to the *Nicotiana* and *Panax* cp genomes, 18 of the genes in the *S. indicum* cp genome have one or two introns. Of these, *rps12, clpP* and *ycf3* have two introns. The *rps12* gene is a uniquely divided gene with the 5′ end exon located in the LSC region while two copies of 3′ end exon and intron are located in the IR region.

**Table 1:** Genes contained in the *Sesamum indicum* cp genome (total 114 genes).

Category for genes	Group of genes	Name of genes
Self replication	rRNA genes	rrn16(×2), rrn23(×2), rrn4.5(×2), rrn5(×2)
	tRNA genes	30 trn genes (6 contain an intron, 7 in the IR regions)
	Small subunit of ribosome	rps2, rps3, rps4, rps7(×2), rps8, rps11, rps12(*), rps14, rps15, rps16*, rps18, rps19
	Large subunit of ribosome	rpl2*( ×2), rpl14, rpl16*, rpl20, rpl22, rpl23(×2),rpl32, rpl33, rpl36
	DNA dependent RNA polymerase	rpoA, rpoB, rpoC1*, rpoC2
Genes for photosynthesis	Subunits of NADH-dehydrogenase	ndhA*, ndhB*(×2), ndhC, ndhD, ndhE, ndhF, ndhG, ndhH, ndhI, ndhJ, ndhK
	Subunits of photosystem I	psaA, psaB, psaC, psaI, psaJ, ycf3**
	Subunits of photosystem II	psbA, psbB, psbC, psbD, psbE, psbF, psbH, psbI,psbJ, psbK, psbL, psbM, psbN, psbT, psbZ
	Subunits of cytochrome b/f complex	petA, petB*, petD*, petG, petL, petN
	Subunits of ATP synthase	atpA, atpB, atpE, atpF*, atpH, atpI
	Large subunit of rubisco	rbcL
Other genes	Translational initiation factor	infA
	Maturase	matK
	Protease	clpP**
	Envelope membrane protein	cemA
	Subunit of Acetyl-CoA-carboxylase	accD
	c-type cytochrom synthesis gene	ccsA
Genes of unknown functions	Open Reading Frames (ORF, ycf)	ycf1, ycf2(×2), ycf4, ycf15(×2)

One and two asterisks after gene names reflect one- and two-intron containing genes, respectively. Genes located in the IR regions are indicated by the (x2) symbol after the gene name. The *rps12* gene is divided: the 5′-*rps12* is located in the LSC region and the 3′-*rps12* in the IR region.

The overall GC and AT contents of the *S. indicum* cp genome are 38% and 62%, respectively. The AT content of the IR regions (57%) is lower than that of the LSC and SSC regions (64% and 68%, respectively). This low AT content in the IR regions is due to the low AT content of four rRNA genes in the region: *rrn16*, *rrn23*, *rrn4.5*, and *rrn5*. The AT content of the protein-coding regions is 60%. Within protein coding region, the AT content is 53% for the first codon position, 62% for the second position, and 70% for the third position, respectively (Table 2). The *Sesamum indicum* cp genome contains 30 tRNA genes that interact with 20 amino acids. Six of the 30 tRNA genes (*trn*K-UUU, *trn*G-UCC, *trn*L-UAA, *trn*V-UAC, *trn*I-GAU and *trn*A-UGC) contain an intron within the anticodon step/loop or D-stem regions.

**Table 2:** Base compositions in the *Sesamum indicum* cp genome.

		T(U)	C	A	G	Sequence lengths(bp)
LSC region		32.5%	18.6%	31.1%	17.8%	85,170
IRa region		28.4%	22.5%	28.2%	20.9%	25,141
IRb region		28.2%	20.9%	28.4%	22.5%	25,141
SSC region		33.8%	17.0%	33.8%	15.5%	17,872
Total		31.3%	19.4%	30.5%	18.8%	153,324
Protein coding genes (CDS)		31.5%	17.6%	30.4%	20.5%	68,097
	1st position	23.0%	18.8%	30.2%	27.5%	22,699
	2nd position	33.0%	20.6%	28.8%	18.0%	22,699
	3rd position	38.0%	13.6%	32.1%	16.1%	22,699

**Figure 2:**
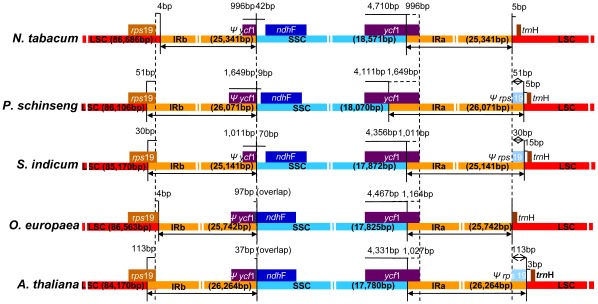
The comparison of the LSC, IR and SSC border regions among five cp genomes.

The length of angiosperm cp genomes is variable primarily due to expansion and contraction of the inverted repeat IR region and the single copy boundary regions. To elucidate this mechanism, the IR/SC boundary regions of the cp genomes of *Sesamum, Nicotiana, Panax, Olea,* and *Arabidopsis* (Figure 2) were compared. *Rps19* and *ycf1* pseudogenes of various lengths were found at the IR/LSC and IR/SSC boundaries, respectively. The *rps19* pseudogene was not found at the LSC region in the *Nicotiana* and *Olea* cp genomes. In the *Sesamum* cp genome, the IR extended into the *rps19* gene and created a short *rps19* pseudogene of 30 bp at the IR/LSC border. This same pseudogene was 51 bp and 113 bp, respectively, in the *Panax* and *Arabidopsis* cp genomes. At the IR/SSC border of the *Sesamum* cp genome, the IR extended into the *ycf1* gene to create a long *ycf1* pseudogene of 1,100 bp at the IR/LSC border. This *ycf1* pseudogene was 1,164 bp in *Olea* and 1,649 bp in *Panax*. In addition, the *ycf1* pseudogene and the *ndh*F gene overlap in both the *Olea* and *Sesamum* cp genomes for 97 and 70 bp, respectively.

A comparison of base substitutions and indels in the cp genomes of *Sesamum, Olea*, *Nicotiana* and *Panax* was conducted. The average sequence divergence of the IR regions is 0.91% between *Sesamum* and *Olea*, 1.52% between *Sesamum* and *Nicotiana*, and 1.67% between *Sesamum* and *Panax*. The divergence values of the LSC regions are 3.23%, 5.64% and 6.17%, respectively, while the divergence values of the SSC regions are 7.06%, 11.25%, 11.98%, respectively. The detailed sequence comparisons in each gene coding region among *Sesamum*, *Olea*, *Nicotiana*, and *Panax* are provided in supplemental data (Table S1, S2, and S3). The average Ka/Ks ratios were 0.73, 0.63 and 0.61 in the IR region; 0.23, 0.17 and 0.27 in the LSC region; and 0.40, 0.35 and 0.41 in the SSC region. We also compare the sequence divergence according to the functional groups of genes. The rRNA gene group in the IR region showed the most conserved nature. In contrast, the *matK, ccsA, accD, ycf(5), infA,* and *cemA* genes exhibit high sequence divergences. *Rps*16 and *rpl*33 genes showed a Ka/Ks ratio greater than 1.00 in *Sesamum* and *Olea* comparision.

**Figure 3:**
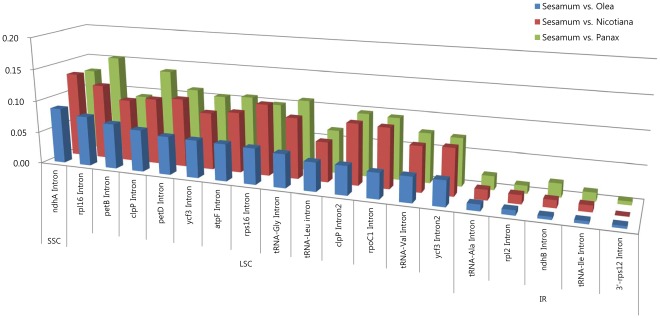
The comparisons of 19 intron regions of the chloroplast genomes in the three different comparisons of *Sesamum vs. Olea*, *Sesamum vs. Nicotiana*, and *Sesamum vs. Panax*. Y axis indicates the sequence divergences.

*Sesamum* cp genome contain 128 intergenic spacer (IGS) regions which longer than 10 bp in length. The indel and base substitution patterns of the IGS regions were compared among the four cp genomes (Table S1, S2, and S3). The sequence divergences of IGS regions of *Sesamum* and *Olea* ranged from 0.00% to 11.67% in the IR region, 0.00% to 23.18% in the LSC region, and 0.00% to 13.69% in the SSC region, respectively. The divergence values between *Sesamum* and *Panax* ranged from 0.00% to 7.55% in the IR region, 0.00% to 31.52% in the LSC region and 0.00% to 23.91% in the SSC region, respectively. The divergence values between *Sesamum* and *Nicotiana* ranged from 0.00% to 11.11% in the IR region, 0.00% to 50.00% in the LSC region, and 0.00% to 24.55% in the SSC region, respectively. The sequence divergence patterns in the 19 intron regions are provided in Figure 3.

**Figure 4:**
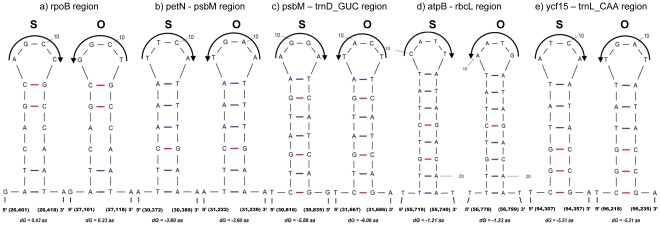
Small inversion mutations and associated secondary structures between the cp genomes of *Sesamum* (S) and the cp genome of *Olea* (O).

Upon comparison with the *Olea* cp genome, five short inversions that were associated with inverted sequences were identified in the *Sesamum* cp genome (Figure 4). These five regions form distinct stem-loop hairpin structures, and the sequence orientation is opposite in the two chloroplast genomes at the loop regions. The first short inversion is located on the *rpoB* coding region, genome coordinates 26,401 bp–26,418 bp (Figure 4a). The other four small inversions are located on the inter-genic spacers (Figure 4b–4e). The small inversion regions correspond to the stem-loop-forming regions located downstream of the genes involved in stabilizing mRNA molecules. Large inversion mutations have been frequently noted in several widely diverse vascular plants [24], [25], [26], [27], [28]. In contrast, the short inversions have been recently reported in just a few cp genomes [29], [30], [31], [32].

Simple sequence repeats (SSRs), also called microsatellites, are considered valuable molecular markers for population genetics because they exhibit high variation within the same species [33], [34]. SSRs are stretches of one to six nucleotide units repeated in tandem and randomly spread throughout cp genomes. SSRs are highly polymorphic due to a high mutation rate that affects the number of repeat units. Within the *Sesame* cp genome, 28 different SSR loci are repeated more than 10 times (Table 3). Of these, 21 loci are homopolymers, four are di-polymers, and three are tri-polymers. Eighteen of the homopolymer loci contain multiple A or T nucleotides, while the other three homopolymer loci contain multiples of C or G nucleotides. All of the di-polymer loci contain multiple AT or TA. These SSR loci contribute to the A-T richness of the cp genome of *Sesamum*. Twenty-three SSR loci occur in the intergenic spacers, while only five are located in the gene coding regions of *atpB*, *rpoC2*, *psbC* and *ycf1*.

**Table 3:** Distribution of simple sequence repeat (SSR) loci in the *Sesamum indicum* cp genome.

Base	Length	No. SSRs	Coodinated Basepairs*
**A**	10	6	239−248, 4,381−4,390, 8,578−8,587, 72,464−72,473, 121,267−121,276, 135,512−135,521
**C**	10	2	51,903−51,912, 66,525−66,534
**C**	11	1	36,699−36,709
**T**	10	11	**13,440**−**13,449, 18,896**−**18,905(rpoC2),** 43,915−43,924, 44,799−44,808, 49,156−49,165, 55,417−55,426(*atpB*), 59,974−59,983, 71,246−71,255, 102,974−102,983, 113,873−113,882, 126,393−126,402(*ycf1*)
**T**	11	1	81,161−81,171
**AT**	10	1	20,268−20,277(*rpoC2*)
**AT**	12	1	42,984−42,995
**TA**	10	1	31,905−31,914
**TA**	12	1	**47,040**−**47,051**
**ATA**	12	1	55,471−55,482
**TTA**	12	1	23,018−23,029
**TTC**	12	1	35,684−35,695(*psbC*)

The coordinated basepairs are the nucleotide number positions starting at the IRa/LSC junction (Figure 1). The underline represents the SSR in the CDS and the bold numbers represent the shared SSR with Olea chloroplast genome.

The coordinated basepairs are the nucleotide number positions starting at the IRa/LSC junction (Figure 1). The underline represents the SSR in the CDS and the bold numbers represent the shared SSR with *Olea* chloroplast genome.

Repeats of 26 bp or longer and with sequence identity greater than 90% were also examined. The majority of these were tandem repeats. The repeating unit, repeating time, repeating location, and the total repeating length of the long repeats were evaluated using the Tandem Repeat Finder. From this analysis, seven total repeats were identified and located. This included two direct tandem repeats, two direct inverted repeats, and three palindromic dispersed repeats as possible gene introduction sites (Table 4). The repeating units are repeated two to four times. One dispersed repeat occurs in the widely separated IR and SSC regions of the *Sesamum* cp genome (Table 4).

**Table 4:** Distribution of large repeat loci in the *Sesamum indicum* cp genome.

Repeat Number	Size (bp)	Repeat	Location	Repeat Unit	Region
**1**	72	direct	CDS(*ycf2*)	GATATTGATGATAGTGAC (4×)	IRb,a
**2**	44	palindromic	**IGS(** ***psbT*** **, ** ***psbN*** **)**	TTGAAGTAATGAGCCTACCAATATAGGTAGGCTCATTACTTCAA	LSC
**3**	41	palindromic dispersed repeats	IGS(*rps12*,*trnV-GAC*), Intron(*ndhA*), IGS(*trnV-GAC*,*rps12*)	TACAGAACCGTACATGAGATTTTCACCTCATACGGCTCCTC	IR,SSC
**4**	33	palindromic	IGS(*petN*, *psbM*)	CTAAGAGATAGATAGTATGGTAGAAAGA	LSC
**5**	30	palindromic dispersed repeats	**CDS(** ***trnS-GCU*** **), CDS(** ***trnS-GGA*** **)**	ACGGAAAGAGAGGGATTCGAACCCTCGGTA	LSC
**6**	30	palindromic dispersed repeats	CDS(*ycf1*)	GGAAGAAAGGGTGGAAAGTGA (2×)	IRb,a
**7**	26	direct	CDS(*ycf2*)	CATCAATATCGTCACTAT (2×)	IRb,a

The repeat units larger than 22 bp are presented in this table and the locations are presented on the Figure 1. The underline represents the SSR in the CDS and the bold numbers represent the shared SSR with *Olea.*

### Phylogenetic Analysis of *Sesamum* Based on the Complete cp Genome Sequences

In order to identified the phylogenetic position of *Sesamum* within the asterid lineages, 32 complete cp genome sequences are downloaded from the Genbank of NCBI database. Two additional eudicot cp genome sequences from *Spinacia* and *Arabidopsis* also included in the phylogenetic analysis as outgroup taxa. The 24 of 32 complete cp DNA sequences are concentrated in the four major families of asterids such as Solanaceae(7), Oleaceae(6), Apiaceae(6), and Asteraceae(5). Other seven sequences represent Convolvulaceae (*Ipomaea*), Pedaliaceae (*Sesamum*), Rubiaceae (*Coffea*), Araliaceae (*Panax* and *Hydrocotyle*), Goodeniaceae (*Scaevola*) and Campanulaceae (*Trachelium*), respectively. We aligned all protein coding gene sequences and four *rrn* gene sequences in a single data matrix. All *trn* genes are excluded in alignment. The aligned data matrix consists of 83,072 bp in length. About 46% of sites are constant, while the other 54% of sites are variable in sequences or indels.

**Figure 5:**
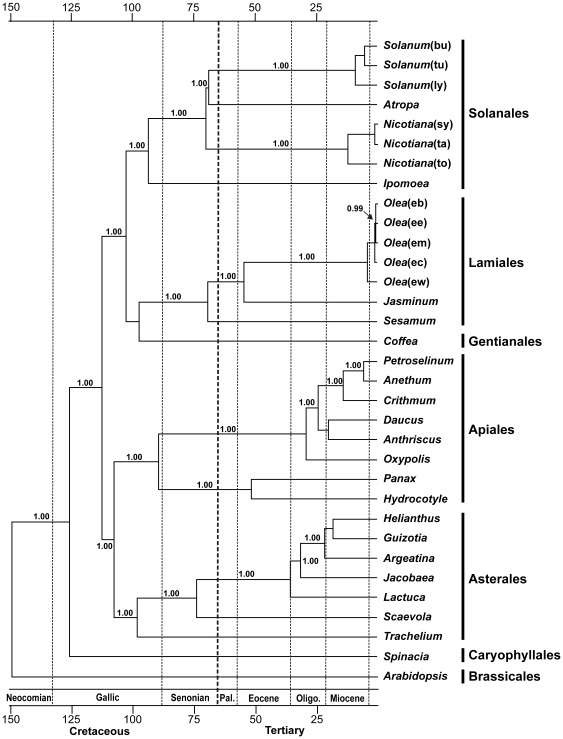
A maximum likelihood tree (-lnL = 428640.9970) of the asterid clade of angiosperm using whole chloroplast genome sequences. The tree was polarized by two outgroup taxa, *Spinacea* and *Arabidopsis*. The GTR+G+I base substitution model was adopted based on the Modeltest. Molecular clock was calibrated using two internal splitting points of the members of Araliaceae (70 mya) and Solanaceae (53 mya). The numbers above each node indicate the Bayesian support percentages. Taxon abbreviations are Solanum(bu): *Solanum bulbocastanum,* Solanum(tu): *Solanum tuberosum*, Solanum(ly): *Solanum lycopersicum*, Nicotiana(sy): *Nicotiana sylvestris*, Nicotiana(ta): *Nicotiana tabacum*, Nicotiana(to): *Nicotiana tomentosiformis*, Olea(eb): *Olea europaea cv. bianchera*, Olea(ee): *Olea europaea* subsp. *europaea*, Olea(em): *Olea europaea* subsp. *maroccana*, Olea(ec): *Olea europaea* subsp. *Cuspidate* and Olea(ew): *Olea europaea* subsp. *Woodiana,* respectively.

A maximum likelihood tree was obtained with the likelihood value of -lnL = 428640.9970 with the GTR+G+I base substitution model (Figure 5). The majority of clades are supported by the high levels of Bayesian percentages. We also estimated the splitting times of major clades of asterids using molecular clocks. Two internal fossil data (Araliaceae 70 million years ago (mya) and Solanaceae 53 mya) were used to calibration the clock [35], [36]. The resulting tree indicate that *Sesamum* (Pedaliaceae) form a sister group to the Oleaeaeae (represented by *Jsaminum* and *Olea*) and the two lineages diverged at the Cretaceous-Tertiary (K-T) boundary in geological time (Figure 5).

## Discussion

### Comparative Analysis of the cp Genomes’ Structure and Gene Order

*Sesamum indicum* is an important oilseed crop that is cultivated worldwide for its high quality edible oil. Approximately 170 completed cp DNA sequences have been reported (NCBI GenBank). Of these, 100 complete cp genomes have been sequenced from various groups of seed plants; however, most of these sequences are concentrated in economically important plant families such as Solanaceae, Poaceae, and Asteraceae. For example, of the 24 complete cp genomes published in Asterids, nine are from the Solanaceae family. In contrast, only three complete cp genome sequences have been reported from Lamiales, and no complete cpDNA sequences have been reported for Lamiaceae *s.l.,* which includes *Sesamum*. The availability of the complete cp DNA sequences from *Sesamum* provides us an improved evolutionary understanding of the chloroplast genome itself and it also serves as an agronomic improvement tool. The complete chloroplast genome of *S. indicum* is 153,324 bp long with an LSC region of 85,170 bp, a SSC region of 17,872 bp, and two IR regions of 25,141 bp each (Figure 1). Overall, the genome order, the genome size, the gene and intron contents, and the AT compositions of the *Sesamum* cp DNA show the characteristics typical of land plant cp genomes (Tables 1–2); however, the IR expansion/contraction in the *Sesamum* cp genome generates slightly different pseudogenes in the boundaries (Figure 2). This is not unusual as slight IR expansion/contraction is relatively common in other cp genomes [37], [38].

### Analysis of Evolutionary Constraints in the *Sesamum* cp Genome

The slow rate of nucleotide substitution in protein-coding genes is a primary reason for the use of chloroplast genes in plant phylogenetic research at higher taxonomic levels [39], [40]. The nucleotide substitution rates in the intergenic spacer and intron regions are higher than the coding sequence (CDS) regions [41], [42]. Such differences in evolution rates are dependent on the sequence and the gene functions. In addition, several previous studies have reported evolutionary differences in cp DNA sequences related to the structural constraints imposed on the plant cp genomes [43]. Most land plant cp genomes include two identical copies of inverted repeat regions. The frequent intra-chromosomal recombination events between these two IR regions of the cp genome provide selective constraints, both on sequence homogeneity and on structural stability [27], [44]. Therefore, the IR region exhibits slower nucleotide substitution rates in comparison to the SSC and LSC regions.

**Figure 6:**
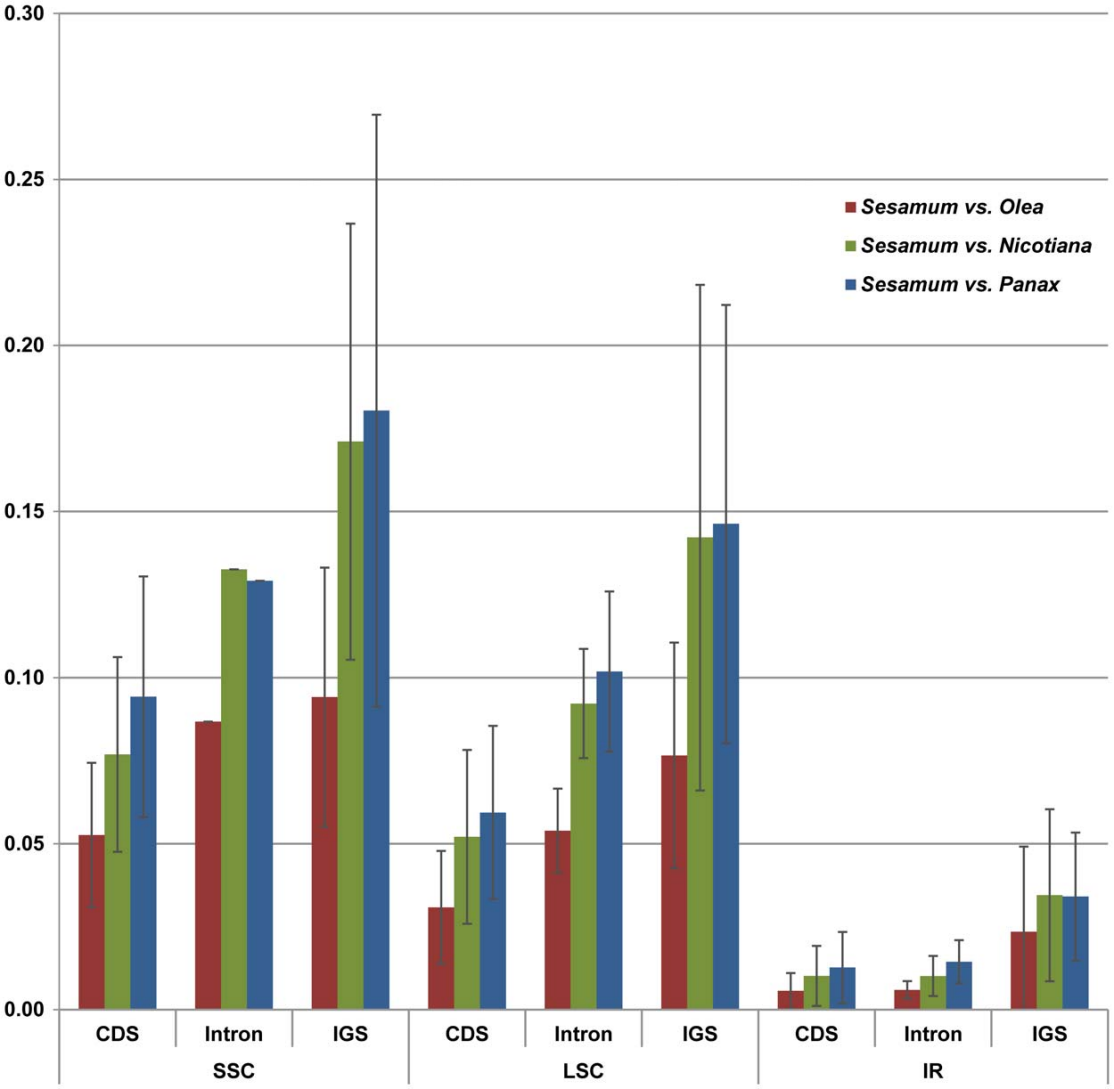
The levels of evolutionary divergences among the SSC, LSC, and IR regions of cp genomes . Y-axis represents the sequence divergences. The IR region evolves slower than the SSC or the LSC regions regardless the CDS, intron and IGS.

To address the evolutionary constraint issue in the *Sesamum* cp genome, a series of comparative sequence analyses were conducted using *Sesamum* cp DNA sequences along with the published cp genome sequences of *Olea, Nicotiana* and *Panax* (Table S1, S2, and S3). These three sequences were selected because they belong to the same or closely related taxonomic orders, Lamiales and Solanales. The gene order of the cp genomes was co-linear among these four genera. An alignment of the protein-coding genes, introns, and intergenic spacer regions, along with positional information of the cp genomes for *Sesamum* and three other genera was performed. Of 114 genes, 84 CDS were analyzed. The 30 tRNA genes were excluded in this comparative analysis due to their short length. A total of 110 IGS and 19 intron sequences were also analyzed. First, the sequence comparison data was partitioned into CDS, intron, and IGS regions. The sequence divergence ratios among the three regions (CDS:intron:IGS) were 1∶1.3∶2.2 between *Sesamum* and *Olea*, 1∶1.3∶2.3 between *Sesamum* and *Nicotiana*, and 1∶1.3∶2.1 between *Sesamum* and *Panax* (Table 5). The ratios in these three comparisons are similar. This clearly suggests that the intron sequences have evolved more rapidly than the CDS but slower than the IGS sequences. Second, the sequence comparison data was partitioned into IR, LSC and SSC regions. The sequence divergence ratios among the three regions (IR:LSC:SSC) were 1∶4.7∶6.9 between *Sesamum* and *Olea*, 1∶5.3∶6.8 between *Sesamum* and *Nicotiana*, and 1∶5.2∶6.2 between *Sesamum* and *Panax*. That the ratios are relatively consistent between three different comparisons clearly suggests that the IR regions have evolved much slower than the LSC and SSC regions (Figure 6). The same tendencies are prominent even when comparing the CDS or noncoding sequences for each of the three regions separately. As an example, 19 intron sequences show markedly slow down patterns of base substitutions in IR regions (Figure 3). Furthermore, the Ka/Ks ratio data for the CDS also indicate that the IR region has stronger selection pressures than either the LSC or SSC regions; therefore, these data confirm that positional effects are stronger constraints for sequence evolution than the functional groups of chloroplast genes.

**Table 5:** Comparisons of protein coding genes (CDS), introns, and intergenic spacers (IGS) at the IR, LSC, and SSC regions of the chloroplast genomes.

		Sesame/Olea							Sesame/Nicotiana							Sesamum/Panax						
Region		NG	LD (IE)	NP	ND	Ks	Ka	Ka/Ks	NG	LD (IE)	NP	ND	Ks	Ka	Ka/Ks	NG	LD (IE)	NP	ND	Ks	Ka	Ka/Ks
CDS	LSC	62	60 (22)	1417	0.0323	0.0815	0.0189	0.23	62	-306 (22)	2471	0.0564	0.1686	0.0280	0.17	62	-21 (21)	2711	0.0617	0.1511	0.0404	0.27
	IR	12	-536 (17)	127	0.0091	0.0113	0.0082	0.73	12	-659 (17)	212	0.0152	0.0270	0.0170	0.63	12	-187 (17)	233	0.0167	0.0296	0.0182	0.61
	SSC	12	-213 (30)	1004	0.0706	0.1398	0.0558	0.40	12	-393 (30)	1599	0.1125	0.2534	0.0878	0.35	12	-330 (30)	1699	0.1198	0.2447	0.1010	0.41
	TOTAL	86	-689 (69)	2548	0.0353	0.0660	0.0276	0.42	86	-1358 (69)	4282	0.0595	0.1630	0.0388	0.24	86	-538 (68)	4643	0.0644	0.1502	0.0497	0.33
Intron	LSC	13	-8	492	0.0546	-	-	-	13	-309	836	0.0939	-	-	-	13	-288	922	0.1035	-	-	-
	IR	5	-4	22	0.0061	-	-	-	5	347	35	0.0106	-	-	-	5	17	54	0.0149	-	-	-
	SSC	1	-18	92	0.0868	-	-	-	1	-68	141	0.1326	-	-	-	1	57	128	0.1292	-	-	-
	TOTAL	19	-30	606	0.0442	-	-	-	19	-30	1012	0.0763	-	-	-	19	-214	1104	0.0817	-	-	-
IGS	LSC	81	-1425	2416	0.0826	-	-	-	81	-675	3982	0.1462	-	-	-	82	-221	4074	0.1494	-	-	-
	IR	19	-117	106	0.0202	-	-	-	19	99	180	0.0337	-	-	-	19	-101	212	0.0399	-	-	-
	SSC	12	126	105	0.1401	-	-	-	12	-397	610	0.2039	-	-	-	12	-627	571	0.2082	-	-	-
	TOTAL	112	-1416	326	0.0760	-	-	-	112	-973	4772	0.1343	-	-	-	113	-949	4857	0.1379	-	-	-
TOTAL		217	-2135	3480	0.0487	-	-	-	217	-2361	10066	0.0901	-	-	-	218	-1701	10604	0.0946	-	-	-

This is a summary table of each calculation from three different comparisons of *Sesamum vs.Olea*, *Sesamum vs. Nicotiana*, and *Sesamum vs. Panax*. The *rps 12* gene is included in the LSC region. Abbreviations: NG, The numbers of genes; LD, the length differences; IE, the indel events; NP, the numbers of polymorphic sites; ND, the nucleotide differences; Ks, the synonymous substitution differences; and Ka, the nonsynonymous substitution differences.

Previous research has indicated that in cp genomes, the IR regions are more conserved than the single copy regions [27], [44], [45]. Between two strands of homologous IR sequences, recombination events occur frequently and successive base collection mechanisms break out; therefore, the base substitution rate in the IR region is slower than that of the LSC and SSC regions [27], [44]. In this report, the cp DNA data was partitioned into two different categories: functional constraints and regional constraints (or positional effects). Data indicate that the regional constraints strongly affect the sequence evolution of cp genomes, while the functional constraints weakly affect the sequence evolution (Figures 3 and 5). Fewer indel events also occur in the IR regions than in the LSC or SSC regions [46].

**Figure 7:**
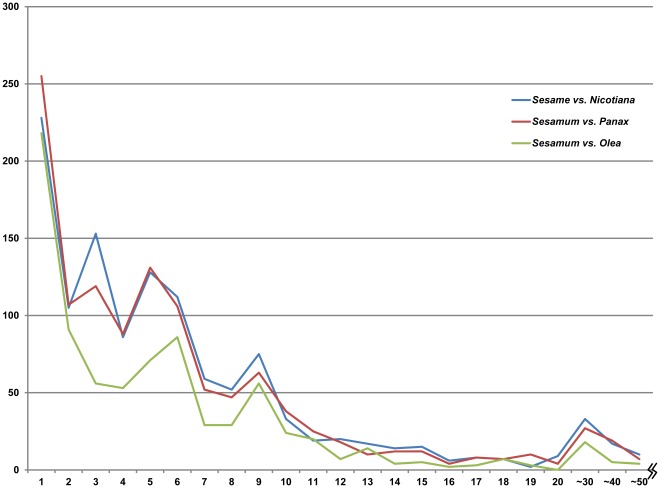
The correlation pattern of indel numbers and indel sizes among three cp genomes. The X-axis and Y-axis represent the indel sizes in base pair and indel numbers, respectively.

The indel patterns of chloroplast genomes from the three different hierarchical comparisons are summarized in Figure 7. The data suggest that similar indel patterns are observed, regardless of the taxonomic hierarchies. The data also indicate that large indels are relatively rare and that the majority of indels are less than 10 bp in length (Figure 7, Table S4).

### Possible Implications for Chloroplast Engineering

The large repeats (26 bp or longer) that exhibited a sequence identity greater than 90% were examined. Many of these repeats contain overlapping components at the same location within the cp genome; therefore, the repeating unit, repeating time and repeating location were determined using REPuter program [47]. Ultimately, seven total repeats were identified and localized (Figure 1). These included two direct repeats, two direct inverted repeats, and three palindromic dispersed repeats as possible gene introduction sites (Table 4). These genes are repeated two to four times. Repeat no. 3, which occurs in three different regions of the cp genome including the two IR and the SSC regions, may have limited utility as a site-specific recombination site. Repeats No. 4 and 5 also show similar challenges for use as vector sites. Two different palindromic repeats are located in the intergenic spacers of the LSC region between *psbT* and *psbN* and between *petN* and *psbM.* These repeats may be useful for the development of site-specific recombination sites for foreign gene cassettes. Two additional useful direct repeats are located on the CDS of the *ycf2* gene in the IR regions. In *Sesamum*, this *ycf2* gene is 6,294 bp long, has an unknown function, and is tolerant of large indel mutations. One direct repeat is especially important because it is 81 bp long and will easily accommodate site-specific recombination. As a result, the two direct and two palindromic repeats present possible foreign gene introduction sites. Three of the seven large repeat loci in the *Sesamum* cp genome are also conserved in the *Olea* cp genome.

In recent years, the universal vector located in the *trnA/trnI* IGS region has been used as a gene introduction site for cp genome engineering [48]. However, it can only be used for plants that are closely related and show high levels of genome sequence homology. This vector has limited utility if the sequences are substantially different; therefore, a species-specific cp vector is expected to be more reliable for plant gene transformation [20]. The complete cp genome sequences are required for the development of site-specific chloroplast vector sites. The genes related to lipid biosynthesis will be primary target genes for alteration in *Sesamum*. The *ACP desaturase* (*SAD*) and *FAD2* genes have been used to produce sunflower [49], [50], [51] and soybean [52], [53] transgenic plants that exhibit oil modification. These genes could be similarly modified in *Sesamum*. The two genes, along with other genes involved in lipid biosynthesis, can be engineered in a single cassette for introduction into the *Sesamum* cp genome. The direct or palindromic repeat sites of the *Sesamum* cp genome represent potential cassette introduction sites that could be used in the development of a sesame-specific chloroplast vector, similar to the *trnA/trnI* flanking sequences used in the universal cp vectors for Solanaceous plants.

### Utility of Repeat Units and cp SSRs

The function and origin of SSRs within the chloroplast genome are not yet fully understood; however, SSR loci are typically present in plant cp genomes and can provide useful information concerning plant population genetics [54], [55]. The presence of SSRs in cp genomes was initially reported in *Pinus radiata* and *Oryza sativa* [34], [56], [57]. Later, Kim and Lee also reported 18 SSR loci and 29 SSR loci in the cp genomes of *Panax* and *Nicotiana*, respectively [58].

Twenty-eight SSR loci were identified in the *Sesamum* cp genome (Table 3). Of these, 21 are homopolymers, four are di-polymers, and three are tri-polymers. Of the homopolymer loci, 18 are composed of A or T multiples, while only three are composed of C or G multiples. All of the di-polymer loci are composed of multiples of AT or TA. Three SSR loci were identical to loci in the *Olea* cp genome. Length variations in SSR loci serve as useful markers for identifying varieties of crops and population genetics [34], [59], [60], [61]. *Sesamum indicum*, which is widely cultivated, has nearly 3,000 cultivars. Using breeding and selection approaches, over 38,000 genetic lines have been developed (United States Department of Agriculture, 2010). Cultivars are distinguished by capsule numbers per node; locule numbers within a capsule; stem branching patterns; seed shapes and colors; flower colors; leaf margin shapes; plant height; trichomes on the fruit, stem, and leaf; fruit maturation; and more [62], [63], [64]; however, many cultivars are difficult to distinguish using these morphological characters. If the cp SSR information is compiled, these SSR loci can provide useful identification tools for some of these cultivars. The complete cp DNA sequences of *Sesamum indicum*, as well as the SSR loci information, provide invaluable sources for developing primers to study specific SSR loci.

### Phylogenetic Position and Origin of *Sesamum* (Pedaliaceae)

Complete cp genome sequences provide rich sources of phylogenetic information. Therefore, several recent phylogenetic studies based on the complete cp genome sequences are addressed successfully for the phylogenetic issues of angiosperm [12], [13]. These genome based analyses across whole angiosperm lineages usually used 61–81 protein coding sequences to assembling the data matrix because of the missing genes in some lineages. Previous genome scale analysis included the 18 complete cp genomes from asterid lineages [12], [65]. In this study, however, we aligned 83 genes from 32 complete cp genome sequences which representing 10 families and 5 orders of asterids. Therefore, our analysis represents the most comprehensive data from asterids. Our phylogenetic tree almost identical to the Angiosperm Phylogeny Group (APG: http://www.mobot.org/mobot/research/apweb) tree and represent the subset of the APG tree. *Sesamum* (Pedaliaceae) form a sister group to the *Olea* and *Jasminum* (Oleaceae) clade (Figure 5). Oleaceae usually positioned as a basal sister family to other Lamiales families [38]. Therefore, our complete cp genome sequences of *Sesamum* represent the core lineage of Lamiales families. The data will be served as a reference sequence for the future genome scale phylogenetic study of this problematic group.

Two major lineages of asterids, asterid I and II, diverged between 114.3±6.7 million years ago (mya) in our tree (Figure 5). This time estimation is highly comparable to the 117–107 mya of the previous reports [35], [66]. Three major orders (Lamiales, Solanales and Gentianales) of asterid I lineages were diversified between 104.2–98.8 mya and it also comparable to the previous estimations of 95±12 mya [67], [68]. Finally, our tree also suggests that the splitting time of Oleaceae (represented by *Jasminum* and *Olea*) and the core Lamiales (represented by *Sesamum*) were approximately 70.1±5.5 mya (Figure 5). It corresponds to the K-T boundary of geological time scale.

## Materials and Methods

### Plants Materials and cpDNA Isolation

Thirty *Sesamum indicum* L. cv. Ansanggae (a black-seeded cultivar) plants were cultivated from seeds originating from a single seed pod of a mother plant. Approximately 100 grams of fresh leaves were harvested from the 30 mature individuals, and two voucher specimens were deposited in the Korea University Herbarium (KUS). To remove starch and sugar from the cells, the fresh leaves were kept in the dark for 48 hrs at 0°C prior to organelle isolation. The leaf tissues were ground using a conventional blender and Sorbitol/TE isolation buffer (0.35 M sorbitol, 50 mM Tris-HCl, 5 mM EDTA, pH 8.0, 0.1% BSA, 0.1% 2-mercaptoethanol). The homogenate is filtered through two layers of miracloth (Calbiochem) and centrifuged at 1,000 g for 15 min at 4°C. The intact cp organelles were purified using sucrose step gradient centrifugation [69]. High purity cp organelles were obtained from the 52–30% sucrose interface. Cp organelles were collected from a total of 12 sucrose gradient tubes in 50 ml volumes. After the careful washing the cp organelles in wash buffer (0.35 M sorbitol, 50 mM Tris-HCl, 5 mM EDTA, pH 8.0, 0.1% BSA), cpDNA was isolated from lysed chloroplasts using ultracentrifugation in a cesium chloride/ethidium bromide gradient. Impurities were removed by dialysis. CpDNAs (Plant DNA Bank of Korea accession number 1996-0001) were quantified using NanoDrop spectrophotometers (Thermo Scientific, Nanodrop 2000), and the cpDNA quality was analyzed on a 1% agarose gel following *BamH*I and *Sac*I restriction enzyme digestion.

### PCR Amplification and Sequencing

Chloroplast DNA sequences were analyzed using the GS-FLX pyrosequencing method [70] and the Genome Sequencer FLX system (Roche, Basal, Switzerland). A total of 133,533 reads, with an average read length of 236 bp, were analyzed to generate 31,540,819 bp of sequence. Because of the contamination of nuclear and mitochondrial DNA in cpDNA, we filtered all reads by extensive BLAST searches using the reference cpDNA sequences from *Panax* [58]. The filtered sequences were assembled using the Newbler program (Roche Diagnostics Company). The combination of the high purity cp organelle isolation procedure and extremely high sequence coverage enabled the assembly of contigs that nearly spanned the entire cp genome. Using BLAST comparisons (BLASTN, PHI-BLAST and BLASTX), we identified 155 contigs that is cp DNA sequences. Of these, three large contigs (85,165 bp, 25,137 bp, 17,877 bp) corresponded to the LSC, IR and SSC regions of the *Sesamum* cp genome, respectively. An additional 152 short contigs were included in the three large contigs. The total length of the contigs was 257,427 bp with an average contig size of 1,660 bp. Gaps between the three large contigs were filled via direct sequencing of PCR products amplified using primers that were complementary to the end sequences of each contig. The amplified regions corresponded to the IR/SSC and IR/LSC boundaries. The sequenced fragments were assembled using Sequencher 4.8 (Gene Code Corporation, Ann Arbor, MI, USA).

### Chloroplast Gene Annotation and Sequence Analyses

Gene annotations and comparative analysis were performed using the BLAST (BLASTN, PHI-BLAST, BLASTX) ORF finder program from the National Center for Biotechnology Information (NCBI) and DOGMA [71]. The nomenclature of cp gene is follows the Chloroplast Genome Database (http://chloroplast.cbio.psu.edu). Codon usage and A-T contents were analyzed using MEGA4 (version 4.1) [72]. Repeating sequences were analyzed using REPuter [47] and further analyzed with the Tandem Repeats Finder, ver. 4.0 [73]. Twenty-eight SSR loci were identified in the *Sesamum* cp genome (Table 3). All SSR regions are PCR amplified and re-sequenced manually in order to prevent the error in pyrosequencing procedure. For sequence comparisons, the gene, intron, and gene spacer regions from the cp genomes of different species were aligned using Clustal X 2.0 [74] and adjusted by hand. Several spacer regions were aligned using MUSCLE [75]. mVISTA were used to compare similarities among different chloroplast genomes [76]. Nucleotide diversity and Ka/Ks value were analyzed using DnaSP (version 4.50) [77]. Secondary structure predicted by mFOLD [78] and TRNAscan-SE [79].

### Phylogenetic Analysis

Thirty-two complete cp DNA sequences representing the asteroid lineage of angiosperm were obtained from NCBI databases (Table S5). For the phylogenetic analysis, 83 gene sequences were initially aligned using the Clustal algorithm [74] and then realigned by the MUSCLE program [75]. Maximum likelihood (ML) analysis was performed using PAUP version 4.0b10 [80] with Modeltest [81]. The GTR+G+I base substitution model was adopted. The Bayesian supporting values of all internal nodes were also calculated under the options of rep = 250,000, lset nst = 6, rates = gamma, basefreq = estimate and burnin = 5000. Molecular time estimation was done using the r8s program [82] implementing semiparametric rate smoothing by penalized likelihood.

## Supporting Information

Table S1 Base substitutions and indels between *Sesamum* and *Olea;* **a)** protein coding genes, **b**) intergenic spacer region and **c)** intron region.
(DOC)

Table S2 Base substitutions and indels between *Sesamum* and *Panax;* **a)** protein coding genes, **b**) intergenic spacer region and **c)** intron region.
(DOC)

Table S3 Base substitutions and indels between *Sesamum* and *Nicotiana;* **a)** protein coding genes, **b**) intergenic spacer region and **c)** intron region.
(DOC)

Table S4 The distribution patterns of indel numbers and indel sizes.
(DOC)

Table S5 The list of accession number of the chloroplast genome sequences used in this study.
(DOC)
