# Supplementary material for: Complete Chloroplast Genome Sequences of Important Oilseed Crop Sesamum indicum L
Source: PLoS One. 2012 May 14;7(5):e35872. doi: 10.1371/journal.pone.0035872 (PMC3351433; doi:10.1371/journal.pone.0035872)
Supplement: Table S1 — Base substitutions and indels between Sesamum and Olea; a) protein coding genes, b) intergenic spacer region and c) intron region. (DOC) [file pone.0035872.s001.doc]

Table S1. Base substitutions and indels between *Sesamum* and *Olea.*

a) protein coding genes

| Region | Genes | Size(Sesamum) | Size(Olea) | INDEL | number of INDEL events | Number of polymorphic site | Nucleotide diversity | Synonymous(Ks) | Nonsynonymous(Ka) | Ka/Ks |
| --- | --- | --- | --- | --- | --- | --- | --- | --- | --- | --- |
| LSC & IR | rps12 | 372 | 372 | 0 | - | 0 | 0.0000 | 0.0000 | 0.0000 | - |
| LSC | psbA | 1059 | 1062 | -3 | 1 | 17 | 0.0161 | 0.0603 | 0.0037 | 0.0614 |
| LSC | matK | 1530 | 1578 | -48 | 4 | 107 | 0.0706 | 0.1085 | 0.0650 | 0.5991 |
| LSC | rps16 | 255 | 270 | -15 | 4 | 19 | 0.0748 | 0.0678 | 0.0751 | 1.1077 |
| LSC | psbK | 186 | 186 | 0 | - | 7 | 0.0376 | 0.0520 | 0.0350 | 0.6731 |
| LSC | psbI | 111 | 111 | 0 | - | 3 | 0.0270 | 0.1203 | 0.0000 | 0.0000 |
| LSC | atpA | 1524 | 1524 | 0 | - | 52 | 0.0341 | 0.1274 | 0.0086 | 0.0675 |
| LSC | atpF | 555 | 555 | 0 | - | 7 | 0.0126 | 0.0340 | 0.0069 | 0.2029 |
| LSC | atpH | 246 | 246 | 0 | - | 9 | 0.0366 | 0.1131 | 0.0112 | 0.0990 |
| LSC | atpI | 744 | 738 | 6 | 1 | 18 | 0.0244 | 0.0845 | 0.0071 | 0.0840 |
| LSC | rps2 | 711 | 711 | 0 | - | 23 | 0.0323 | 0.1105 | 0.0127 | 0.1149 |
| LSC | rpoC2 | 4173 | 4173 | 0 | - | 183 | 0.0441 | 0.0865 | 0.0348 | 0.4023 |
| LSC | rpoC1 | 2055 | 2037 | 18 | 2 | 53 | 0.0261 | 0.0944 | 0.0077 | 0.0816 |
| LSC | rpoB | 3213 | 3213 | 0 | - | 90 | 0.0280 | 0.0862 | 0.0126 | 0.1462 |
| LSC | petN | 90 | 90 | 0 | - | 2 | 0.0222 | 0.1019 | 0.0000 | 0.0000 |
| LSC | psbM | 105 | 105 | 0 | - | 3 | 0.0286 | 0.1271 | 0.0000 | 0.0000 |
| LSC | psbD | 1062 | 1056 | 6 | 1 | 23 | 0.0218 | 0.0874 | 0.0037 | 0.0423 |
| LSC | psbC | 1422 | 1425 | -3 | 1 | 40 | 0.0281 | 0.1157 | 0.0037 | 0.0320 |
| LSC | psbZ | 189 | 189 | 0 | - | 8 | 0.0423 | 0.1152 | 0.0214 | 0.1858 |
| LSC | rps14 | 303 | 303 | 0 | - | 7 | 0.0231 | 0.0801 | 0.0085 | 0.1061 |
| LSC | psaB | 2205 | 2205 | 0 | - | 46 | 0.0209 | 0.0959 | 0.0012 | 0.0125 |
| LSC | psaA | 2253 | 2253 | 0 | - | 48 | 0.0213 | 0.0885 | 0.0029 | 0.0328 |
| LSC | ycf3 | 507 | 507 | 0 | - | 7 | 0.0138 | 0.0482 | 0.0051 | 0.1058 |
| LSC | rps4 | 606 | 606 | 0 | - | 17 | 0.0281 | 0.0838 | 0.0129 | 0.1539 |
| LSC | ndhJ | 477 | 477 | 0 | - | 7 | 0.0147 | 0.0519 | 0.0053 | 0.1021 |
| LSC | ndhK | 702 | 678 | 24 | 1 | 25 | 0.0369 | 0.1272 | 0.0135 | 0.1061 |
| LSC | ndhC | 363 | 363 | 0 | - | 7 | 0.0193 | 0.0804 | 0.0035 | 0.0435 |
| LSC | atpE | 402 | 402 | 0 | - | 18 | 0.0448 | 0.1044 | 0.0296 | 0.2835 |
| LSC | atpB | 1497 | 1497 | 0 | - | 39 | 0.0261 | 0.0970 | 0.0053 | 0.0546 |
| LSC | rbcL | 1434 | 1428 | 6 | 1 | 36 | 0.0252 | 0.0568 | 0.0147 | 0.2588 |
| LSC | accD | 1530 | 1461 | 69 | 4 | 78 | 0.0537 | 0.0944 | 0.0466 | 0.4936 |
| LSC | psaI | 111 | 111 | 0 | - | 2 | 0.0180 | 0.0395 | 0.0119 | 0.3013 |
| LSC | ycf4 | 549 | 555 | -6 | 1 | 24 | 0.0437 | 0.0594 | 0.0413 | 0.6953 |
| LSC | cemA | 690 | 690 | 0 | - | 27 | 0.0391 | 0.1232 | 0.0203 | 0.1648 |
| LSC | petA | 963 | 963 | 0 | - | 31 | 0.0322 | 0.0974 | 0.0149 | 0.1530 |
| LSC | psbJ | 123 | 123 | 0 | - | 4 | 0.0325 | 0.1367 | 0.0000 | 0.0000 |
| LSC | psbL | 117 | 117 | 0 | - | 0 | 0.0000 | 0.0000 | 0.0000 | - |
| LSC | psbF | 120 | 120 | 0 | - | 1 | 0.0083 | 0.0341 | 0.0000 | 0.0000 |
| LSC | psbE | 252 | 252 | 0 | - | 5 | 0.0198 | 0.0945 | 0.0000 | 0.0000 |
| LSC | petL | 96 | 96 | 0 | - | 6 | 0.0625 | 0.1327 | 0.0433 | 0.3263 |
| LSC | petG | 114 | 114 | 0 | - | 0 | 0.0000 | 0.0000 | 0.0000 | - |
| LSC | psaJ | 135 | 135 | 0 | - | 2 | 0.0148 | 0.0632 | 0.0000 | 0.0000 |
| LSC | rpl33 | 201 | 201 | 0 | - | 10 | 0.0498 | 0.0496 | 0.0520 | 1.0484 |
| LSC | rps18 | 306 | 306 | 0 | - | 3 | 0.0098 | 0.0450 | 0.0000 | 0.0000 |
| LSC | rpl20 | 387 | 387 | 0 | - | 16 | 0.0413 | 0.0858 | 0.0305 | 0.3555 |
| LSC | clpP | 591 | 591 | 0 | - | 23 | 0.0389 | 0.0776 | 0.0291 | 0.3750 |
| LSC | psbB | 1527 | 1527 | 0 | - | 36 | 0.0236 | 0.1005 | 0.0026 | 0.0259 |
| LSC | psbT | 108 | 108 | 0 | - | 2 | 0.0185 | 0.0414 | 0.0121 | 0.2923 |
| LSC | psbN | 132 | 132 | 0 | - | 4 | 0.0303 | 0.1433 | 0.0000 | 0.0000 |
| LSC | psbH | 222 | 222 | 0 | - | 6 | 0.0270 | 0.0992 | 0.0060 | 0.0605 |
| LSC | petB | 648 | 648 | 0 | - | 18 | 0.0278 | 0.1218 | 0.0020 | 0.0164 |
| LSC | petD | 483 | 483 | 0 | - | 5 | 0.0104 | 0.0443 | 0.0000 | 0.0000 |
| LSC | rpoA | 1014 | 1014 | 0 | - | 40 | 0.0394 | 0.0894 | 0.0280 | 0.3132 |
| LSC | rps11 | 417 | 417 | 0 | - | 17 | 0.0408 | 0.1435 | 0.0097 | 0.0676 |
| LSC | rpl36 | 114 | 114 | 0 | - | 3 | 0.0263 | 0.1253 | 0.0000 | 0.0000 |
| LSC | infA | 234 | 234 | 0 | - | 12 | 0.0513 | 0.1925 | 0.0168 | 0.0873 |
| LSC | rps8 | 405 | 405 | 0 | - | 24 | 0.0593 | 0.2225 | 0.0195 | 0.0876 |
| LSC | rpl14 | 369 | 369 | 0 | - | 5 | 0.0136 | 0.0608 | 0.0000 | 0.0000 |
| LSC | rpl16 | 408 | 408 | 0 | - | 10 | 0.0245 | 0.0976 | 0.0032 | 0.0328 |
| LSC | rps3 | 663 | 657 | 6 | 1 | 36 | 0.0548 | 0.1606 | 0.0315 | 0.1961 |
| LSC | rpl22 | 468 | 468 | 0 | - | 35 | 0.0763 | 0.1317 | 0.0649 | 0.4928 |
| LSC | rps19 | 279 | 279 | 0 | - | 11 | 0.0394 | 0.1403 | 0.0140 | 0.0998 |
| LSC TOTAL |  | 44127 | 44067 | 60 | 22 | 1417 | 0.0323 | 0.0815 | 0.0189 | 0.2319 |
| IR | rpl2 | 825 | 825 | 0 | - | 7 | 0.0085 | 0.0156 | 0.0064 | 0.4103 |
| IR | rpl23 | 285 | 282 | 3 | 1 | 2 | 0.0071 | 0.0166 | 0.0046 | 0.2771 |
| IR | ycf2 | 6294 | 6834 | -540 | 15 | 93 | 0.0150 | 0.0155 | 0.0147 | 0.9484 |
| IR | ycf15 | 150 | 150 | 0 | - | 2 | 0.0133 | 0.0000 | 0.0000 | - |
| IR | ndhB | 1533 | 1533 | 0 | - | 2 | 0.0013 | 0.0028 | 0.0008 | 0.2857 |
| IR | rps7 | 468 | 468 | 0 | - | 2 | 0.0043 | 0.0183 | 0.0000 | 0.0000 |
| IR | rrn16 | 1491 | 1490 | 1 | 1 | 2 | 0.0013 | - | - | - |
| IR | rrn23 | 2811 | 2811 | 0 | - | 17 | 0.0061 | - | - | - |
| IR | rrn4.5 | 103 | 103 | 0 | - | 0 | 0.0000 | - | - | - |
| IR | rrn5 | 121 | 121 | 0 | - | 0 | 0.0000 | - | - | - |
| IR TOTAL |  | 14081 | 14617 | -536 | 17 | 127 | 0.0091 | 0.0113 | 0.0082 | 0.7257 |
| SSC | ndhF | 2256 | 2226 | 30 | 5 | 154 | 0.0695 | 0.1629 | 0.0495 | 0.3039 |
| SSC | rpl32 | 177 | 162 | 15 | 1 | 10 | 0.0617 | 0.1552 | 0.0332 | 0.2139 |
| SSC | ccsA | 978 | 972 | 6 | 1 | 59 | 0.0607 | 0.1608 | 0.0377 | 0.2345 |
| SSC | ndhD | 1503 | 1530 | -27 | 1 | 76 | 0.0506 | 0.1344 | 0.0295 | 0.2195 |
| SSC | psaC | 246 | 246 | 0 | - | 9 | 0.0366 | 0.1919 | 0.0000 | 0.0000 |
| SSC | ndhE | 306 | 306 | 0 | - | 11 | 0.0359 | 0.1273 | 0.0127 | 0.0998 |
| SSC | ndhG | 531 | 531 | 0 | - | 18 | 0.0339 | 0.1033 | 0.0149 | 0.1442 |
| SSC | ndhI | 507 | 507 | 0 | - | 19 | 0.0384 | 0.0698 | 0.0319 | 0.4570 |
| SSC | ndhA | 1092 | 1086 | 6 | - | 39 | 0.0360 | 0.0938 | 0.0199 | 0.2122 |
| SSC | ndhH | 1182 | 1182 | 0 | - | 39 | 0.0330 | 0.1288 | 0.0098 | 0.0761 |
| SSC | rps15 | 273 | 255 | 18 | 1 | 18 | 0.0706 | 0.2097 | 0.0410 | 0.1955 |
| SSC | ycf1 | 5370 | 5631 | -261 | 21 | 552 | 0.1045 | 0.1717 | 0.0987 | 0.5748 |
| SSC TOTAL |  | 14421 | 14634 | -213 | 30 | 1004 | 0.0706 | 0.1398 | 0.0558 | 0.3991 |
| TOTAL |  | 72629 | 73318 | -689 | 69 | 2548 | 0.0353 | 0.0660 | 0.0276 | 0.4182 |

Table S1. b) intergenic spacer region

| region | IGS | Size(Sesamum) | Size(Olea) | Indel | polymorphic site | nucleotide diversity | nucleotide diversity |
| --- | --- | --- | --- | --- | --- | --- | --- |
| LSC | tRNA-His/psbA | 292 | 447 | -155 | 67 | 0.2318 | 0.02261 |
| LSC | psbA/tRNA-Lys | 239 | 236 | 3 | 15 | 0.0647 | 0.01449 |
| LSC | tRNA-Lys/matK | 708 | 649 | 59 | 36 | 0.0558 | 0.01087 |
| LSC | matK/tRNA-Lys | 256 | 268 | -12 | 11 | 0.0430 | 0.01252 |
| LSC | tRNA-Lys/rps16 | 889 | 896 | -7 | 101 | 0.1229 | 0.03802 |
| LSC | rps16/tRnA-Gln | 1146 | 1652 | -506 | 125 | 0.1215 | 0.03612 |
| LSC | tRNA-Gln/psbK | 341 | 334 | 7 | 14 | 0.0422 | 0.00573 |
| LSC | psbK/psbI | 384 | 417 | -33 | 20 | 0.0531 | 0.03186 |
| LSC | psbI/tRNA-Ser | 120 | 120 | 0 | 3 | 0.0256 | 0.02899 |
| LSC | tRNA-Ser/tRNA-Gly | 703 | 699 | 4 | 64 | 0.0964 | 0.02613 |
| LSC | tRNA-Gly/tRNA-Arg | 188 | 168 | 20 | 14 | 0.0848 | 0.04762 |
| LSC | tRNA-Arg/atpA | 104 | 111 | -7 | 10 | 0.0971 | 0.02941 |
| LSC | atpA/atpF | 55 | 68 | -13 | 4 | 0.0727 | 0.03846 |
| LSC | atpF/atpH | 376 | 359 | 17 | 27 | 0.0761 | 0.02667 |
| LSC | atpH/atpI | 1001 | 1072 | -71 | 70 | 0.0735 | 0.02523 |
| LSC | atpI/rps2 | 230 | 228 | 2 | 13 | 0.0586 | 0.02092 |
| LSC | rps2/rpoC2 | 207 | 208 | -1 | 17 | 0.0850 | 0.01415 |
| LSC | rpoC2/rpoC1 | 154 | 157 | -3 | 9 | 0.0621 | 0.03636 |
| LSC | rpoC1/rpoB | 26 | 26 | 0 | 1 | 0.0385 | 0.00000 |
| LSC | rpoB/tRNA-Cys | 1163 | 1191 | -28 | 94 | 0.0830 | 0.02913 |
| LSC | tRNA-Cys/petN | 823 | 760 | 63 | 71 | 0.0966 | 0.02051 |
| LSC | petN/psbM | 981 | 1164 | -183 | 87 | 0.0919 | 0.02690 |
| LSC | psbM/tRNA-Asp | 523 | 656 | -133 | 42 | 0.0830 | 0.02885 |
| LSC | tRNA-Asp/tRNA-Tyr | 108 | 107 | 1 | 2 | 0.0206 | 0.01835 |
| LSC | tRNA-Tyr/tRNA-Glu | 59 | 59 | 0 | 2 | 0.0339 | 0.00000 |
| LSC | tRNA-Glu/tRNA-Thr | 549 | 777 | -228 | 38 | 0.0712 | 0.02689 |
| LSC | tRNA-Thr/psbD | 1325 | 1322 | 3 | 102 | 0.0792 | 0.02982 |
| LSC | psbD/psbC | - | - | - | - | - | 0.00000 |
| LSC | psbC/tRNA-Ser | 247 | 237 | 10 | 23 | 0.0983 | 0.00481 |
| LSC | tRNA-Ser/psbZ | 334 | 350 | -16 | 22 | 0.0667 | 0.01475 |
| LSC | psbZ/tRNA-Gly | 286 | 297 | -11 | 22 | 0.0791 | 0.05735 |
| LSC | tRNA-Gly/tRNA-fM | 177 | 174 | 3 | 20 | 0.1242 | 0.02273 |
| LSC | tRNA-fM/rps14 | 148 | 145 | 3 | 9 | 0.0629 | 0.01342 |
| LSC | rps14/psaB | 122 | 122 | 0 | 8 | 0.0656 | 0.00814 |
| LSC | psaB/psaA | 25 | 25 | 0 | 0 | 0.0000 | 0.00000 |
| LSC | psaA/ycf3 | 742 | 741 | 1 | 57 | 0.0860 | 0.01707 |
| LSC | ycf3/tRNA-Ser | 864 | 776 | 88 | 71 | 0.0937 | 0.02635 |
| LSC | tRNA-Ser/rps4 | 290 | 320 | -30 | 25 | 0.0862 | 0.02370 |
| LSC | rps4/tRNA-Thr | 351 | 325 | 26 | 32 | 0.0991 | 0.01744 |
| LSC | tRNA-Thr/tRNA-Leu | 694 | 672 | 22 | 57 | 0.0880 | 0.02792 |
| LSC | tRNA-Leu/tRNA-Phe | 341 | 345 | -4 | 30 | 0.0890 | 0.02500 |
| LSC | tRNA-Phe/ndhJ | 671 | 673 | -2 | 68 | 0.1048 | 0.02168 |
| LSC | ndhJ/ndhK | 75 | 105 | -30 | 6 | 0.0800 | 0.01905 |
| LSC | ndhK/ndhC | 53 | 55 | -2 | 0 | 0.0000 | 0.00000 |
| LSC | ndhC/tRNA-Val | 1156 | 1101 | 55 | 108 | 0.1015 | 0.02374 |
| LSC | tRNA-Val/tRNA-Met | 180 | 181 | -1 | 12 | 0.0667 | 0.02778 |
| LSC | tRNA-Met/atpB | 217 | 205 | 12 | 19 | 0.0936 | 0.01523 |
| LSC | atpB/Rbcl | 778 | 785 | -7 | 54 | 0.0712 | 0.01538 |
| LSC | Rbcl/accD | 635 | 694 | -59 | 43 | 0.0720 | 0.01577 |
| LSC | accD/psaI | 687 | 701 | -14 | 44 | 0.0666 | 0.02343 |
| LSC | psaI/ycf4 | 444 | 442 | 2 | 25 | 0.0572 | 0.01720 |
| LSC | ycf4/cemA | 876 | 872 | 4 | 76 | 0.0897 | 0.01231 |
| LSC | cemA/petA | 214 | 244 | -30 | 19 | 0.0896 | 0.01485 |
| LSC | petA/psbJ | 1004 | 970 | 34 | 74 | 0.0825 | 0.01988 |
| LSC | psbJ/psbL | 133 | 133 | 0 | 3 | 0.0226 | 0.01515 |
| LSC | psbL/psbF | 23 | 22 | 1 | 1 | 0.0455 | 0.00000 |
| LSC | psbF/psbE | 14 | 14 | 0 | 1 | 0.0714 | 0.00000 |
| LSC | psbE/petL | 913 | 1181 | -268 | 73 | 0.0817 | 0.01726 |
| LSC | petL/petG | 181 | 172 | 9 | 16 | 0.0952 | 0.01316 |
| LSC | petG/tRNA-Trp | 127 | 121 | 6 | 7 | 0.0579 | 0.00826 |
| LSC | tRNA-Trp/tRNA-Pro | 163 | 170 | -7 | 13 | 0.0798 | 0.01493 |
| LSC | tRNA-Pro/psaJ | 389 | 384 | 5 | 25 | 0.0665 | 0.01558 |
| LSC | psaJ/rpl33 | 475 | 454 | 21 | 32 | 0.0727 | 0.02489 |
| LSC | rpl33/rps18 | 171 | 174 | -3 | 14 | 0.0966 | 0.01807 |
| LSC | rps18/rpl20 | 234 | 239 | -5 | 19 | 0.0812 | 0.02075 |
| LSC | rpl20/rps12-2 | 797 | 799 | -2 | 50 | 0.0629 | 0.01034 |
| LSC | rps12-2/clpP | 536 | 536 | 0 | 11 | 0.0827 | 0.01266 |
| LSC | clpP/psbB | 419 | 451 | -32 | 37 | 0.0896 | 0.00948 |
| LSC | psbB/psbT | 180 | 186 | -6 | 22 | 0.1222 | 0.01887 |
| LSC | psbT/psbN | 60 | 59 | 1 | 4 | 0.0678 | 0.00000 |
| LSC | psbN/psbH | 105 | 105 | 0 | 5 | 0.0476 | 0.00000 |
| LSC | psbH/petB | 124 | 125 | -1 | 9 | 0.0726 | 0.05344 |
| LSC | petB/petD | 188 | 182 | 6 | 12 | 0.0659 | 0.01724 |
| LSC | petD/rpoA | 181 | 183 | -2 | 5 | 0.0284 | 0.03478 |
| LSC | rpoA/rps11 | 71 | 71 | 0 | 6 | 0.0845 | 0.08108 |
| LSC | rps11/rpl36 | 101 | 101 | 0 | 9 | 0.0891 | 0.05505 |
| LSC | rpl36/infA | 95 | 95 | 0 | 3 | 0.0316 | 0.02609 |
| LSC | infA/rps8 | 124 | 121 | 3 | 10 | 0.0826 | 0.03419 |
| LSC | rps8/rpl14 | 180 | 207 | -27 | 14 | 0.0800 | 0.02618 |
| LSC | rpl14/rpl16 | 133 | 114 | 19 | 4 | 0.0351 | 0.06569 |
| LSC | rpl16/rps3 | 152 | 151 | 1 | 16 | 0.1135 | 0.02632 |
| LSC | rpl22/rps19 | 64 | 61 | 3 | 12 | 0.1967 | 0.01370 |
| LSC TOTAL |  | 30894 | 32319 | -1425 | 2416 | 0.0826 | 0.02626 |

Table S1. b) (continued)

| region | IGS | Size(Sesamum) | Size(Olea) | Indel | number of INDEL events | number of polymorphic site | nucleotide diversity |
| --- | --- | --- | --- | --- | --- | --- | --- |
| IR | rps19/rpl2 | 64 | 60 | 4 | 7 | 0.1167 | 0.01887 |
| IR | rpl2/rpl23 | 18 | 18 | 0 | 0 | 0.0000 | 0.00000 |
| IR | rpl23/tRNA-His | 165 | 166 | -1 | 3 | 0.0182 | 0.00588 |
| IR | tRNA-His/ycf2 | 88 | 88 | 0 | 3 | 0.0341 | 0.00000 |
| IR | ycf2/ycf15 | 189 | 201 | -12 | 4 | 0.0212 | 0.00000 |
| IR | ycf15/tRNA-Leu | 359 | 355 | 4 | 16 | 0.0458 | 0.01563 |
| IR | tRNA-Leu/ndhB | 547 | 537 | 10 | 6 | 0.0113 | 0.00173 |
| IR | ndhB/rps7 | 274 | 274 | 0 | 0 | 0.0000 | 0.00000 |
| IR | rps7/rps12-2 | 53 | 53 | 0 | 2 | 0.0377 | 0.00000 |
| IR | rps12-2/tRNA-Val | 1603 | 1596 | 7 | 23 | 0.0147 | 0.00442 |
| IR | tRNA-Val/rrn16 | 227 | 330 | -103 | 6 | 0.0264 | 0.00441 |
| IR | rrn16/tRNA-Ile | 299 | 299 | 0 | 7 | 0.0234 | 0.00000 |
| IR | tRNA-Ile/tRNA-Ala | 64 | 66 | -2 | 1 | 0.0156 | 0.00000 |
| IR | tRNA-Ala/rrn23 | 157 | 147 | 10 | 2 | 0.0136 | 0.00000 |
| IR | rrn23/rrn4_5 | 98 | 98 | 0 | 0 | 0.0000 | 0.01020 |
| IR | rrn4_5/rrn5 | 255 | 255 | 0 | 3 | 0.0118 | 0.00413 |
| IR | rrn5/tRNA-Arg | 243 | 254 | -11 | 4 | 0.0165 | 0.00000 |
| IR | tRNA-Arg/tRNA-Asn | 568 | 590 | -22 | 14 | 0.0246 | 0.00850 |
| IR | tRNA-Asn/ycf1 | 328 | 329 | -1 | 5 | 0.0152 | 0.00306 |
| IR TOTAL |  | 5599 | 5716 | -117 | 106 | 0.0202 | 0.00440 |
| SSC | ndhF/rpl32 | 517 | 478 | 39 | 64 | 0.1342 | 0.02733 |
| SSC | rpl32/tRNA-Leu | 883 | 830 | 53 | 85 | 0.1079 | 0.03141 |
| SSC | tRNA-Leu/ccsA | 91 | 90 | 1 | 10 | 0.1149 | 0.02500 |
| SSC | ccsA/ndhD | 252 | 251 | 1 | 33 | 0.1369 | 0.02727 |
| SSC | ndhD/psaC | 124 | 84 | 40 | 6 | 0.0714 | 0.04274 |
| SSC | psaC/ndhE | 250 | 256 | -6 | 21 | 0.0857 | 0.01210 |
| SSC | ndhE/ndhG | 225 | 201 | 24 | 25 | 0.1276 | 0.02765 |
| SSC | ndhG/ndhI | 347 | 322 | 25 | 33 | 0.1071 | 0.02432 |
| SSC | ndhI/ndhA | 79 | 93 | -14 | 4 | 0.0506 | 0.03659 |
| SSC | ndhA/ndhH | 1 | 1 | 0 | 0 | 0.0000 | 0.00000 |
| SSC | ndhH/rps15 | 97 | 115 | -18 | 9 | 0.0928 | 0.01087 |
| SSC | rps15/ycf1 | 367 | 386 | -19 | 36 | 0.1011 | 0.03325 |
| SSC TOTAL |  | 3233 | 3107 | 126 | 326 | 0.1401 | 0.02774 |
| TOTAL |  | 39726 | 41142 | -1416 | 2848 | 0.0760 | 0.02332 |

*Table S1. c) intron region*

| region | intron | Size(Sesamum) | Size(Olea) | Indel | polymorphic site | nucleotide diversity | nucleotide diversity |
| --- | --- | --- | --- | --- | --- | --- | --- |
| LSC | rps16 | 859 | 875 | -16 | 47 | 0.0554 | 0.01130 |
| LSC | tRNA-Gly | 688 | 678 | 10 | 35 | 0.0518 | 0.02152 |
| LSC | atpF | 695 | 698 | -3 | 39 | 0.0573 | 0.02216 |
| LSC | rpoC1 | 783 | 746 | 37 | 29 | 0.0390 | 0.00927 |
| LSC | ycf3 | 725 | 743 | -18 | 42 | 0.0583 | 0.00936 |
| LSC | ycf3 | 704 | 705 | -1 | 27 | 0.0388 | 0.00978 |
| LSC | tRNA-Leu | 488 | 476 | 12 | 21 | 0.0443 | 0.01183 |
| LSC | tRNA-Val | 578 | 572 | 6 | 22 | 0.0389 | 0.01211 |
| LSC | clpP | 630 | 644 | -14 | 41 | 0.0652 | 0.01741 |
| LSC | clpP | 739 | 809 | -70 | 32 | 0.0441 | 0.01697 |
| LSC | petB | 721 | 705 | 16 | 49 | 0.0706 | 0.01793 |
| LSC | petD | 733 | 730 | 3 | 42 | 0.0595 | 0.01473 |
| LSC | rpl16 | 886 | 856 | 30 | 66 | 0.0781 | 0.01807 |
| LSC total |  | 9229 | 9237 | -8 | 492 | 0.0546 | 0.01488 |
| IR | rpl2 | 667 | 675 | -8 | 5 | 0.0075 | 0.00152 |
| IR | ndhB | 679 | 679 | 0 | 3 | 0.0044 | 0.00147 |
| IR | rps12-2 | 536 | 536 | 0 | 2 | 0.0037 | 0.00373 |
| IR | tRNA-Ile | 950 | 947 | 3 | 4 | 0.0042 | 0.00000 |
| IR | tRNA-Ala | 812 | 811 | 1 | 8 | 0.0099 | 0.00124 |
| IR total |  | 3644 | 3648 | -4 | 22 | 0.0061 | 0.00138 |
| SSC | ndhA | 1080 | 1098 | -18 | 92 | 0.0868 | 0.02081 |
| SSC total |  | 1080 | 1098 | -18 | 92 | 0.0868 | 0.02081 |
| TOTAL |  | 13953 | 13983 | -30 | 606 | 0.0442 | 0.01184 |
