# Supplementary material for: Complete Chloroplast Genome Sequences of Important Oilseed Crop Sesamum indicum L
Source: PLoS One. 2012 May 14;7(5):e35872. doi: 10.1371/journal.pone.0035872 (PMC3351433; doi:10.1371/journal.pone.0035872)
Supplement: Table S2 — Base substitutions and indels between Sesamum and Panax; a) protein coding genes, b) intergenic spacer region and c) intron region. (DOC) [file pone.0035872.s002.doc]

Table S2. Base substitutions and indels between *Sesamum* and *Panax.*

a) protein coding genes

| Region | Genes | Size(Sesamum) | Size(Panax) | INDEL | number of INDEL events | Number of polymorphic site | Nucleotide diversity | Synonymous(Ks) | Nonsynonymous(Ka) | Ka/Ks |
| --- | --- | --- | --- | --- | --- | --- | --- | --- | --- | --- |
| LSC & IR | rps12 | 372 | 372 | 0 | - | 11 | 0.0296 | 0.0903 | 0.0109 | 0.1207 |
| LSC | psbA | 1059 | 1062 | -3 | 1 | 28 | 0.0264 | 0.1162 | 0.0025 | 0.0215 |
| LSC | matK | 1530 | 1512 | 18 | 4 | 192 | 0.1275 | 0.2357 | 0.1147 | 0.4866 |
| LSC | rps16 | 255 | 237 | 18 | 1 | 19 | 0.0802 | 0.2047 | 0.0514 | 0.2511 |
| LSC | psbK | 186 | 186 | 0 | - | 14 | 0.0753 | 0.1367 | 0.0643 | 0.4704 |
| LSC | psbI | 111 | 111 | 0 | - | 5 | 0.0450 | 0.2142 | 0.0000 | 0.0000 |
| LSC | atpA | 1524 | 1524 | 0 | - | 95 | 0.0623 | 0.2398 | 0.0183 | 0.0763 |
| LSC | atpF | 555 | 555 | 0 | - | 30 | 0.0541 | 0.1759 | 0.0258 | 0.1467 |
| LSC | atpH | 246 | 246 | 0 | - | 12 | 0.0488 | 0.2058 | 0.0000 | 0.0000 |
| LSC | atpI | 744 | 744 | 0 | - | 30 | 0.0403 | 0.1716 | 0.0053 | 0.0309 |
| LSC | rps2 | 711 | 711 | 0 | - | 46 | 0.0647 | 0.2323 | 0.0275 | 0.1184 |
| LSC | rpoC2 | 4173 | 4164 | 9 | 7 | 311 | 0.0750 | 0.1432 | 0.0627 | 0.4378 |
| LSC | rpoC1 | 2055 | 2070 | -15 | 3 | 111 | 0.0543 | 0.2000 | 0.0198 | 0.0990 |
| LSC | rpoB | 3213 | 3216 | -3 | 1 | 191 | 0.0594 | 0.2213 | 0.0210 | 0.0949 |
| LSC | petN | 90 | 90 | 0 | - | 3 | 0.0333 | 0.1585 | 0.0000 | 0.0000 |
| LSC | psbM | 105 | 111 | -6 | 1 | 11 | 0.1048 | 0.3166 | 0.0586 | 0.1851 |
| LSC | psbD | 1062 | 1062 | 0 | - | 27 | 0.0254 | 0.1062 | 0.0037 | 0.0348 |
| LSC | psbC | 1422 | 1422 | 0 | - | 68 | 0.0478 | 0.2113 | 0.0055 | 0.0260 |
| LSC | psbZ | 189 | 189 | 0 | - | 9 | 0.0476 | 0.1394 | 0.0215 | 0.1542 |
| LSC | rps14 | 303 | 303 | 0 | - | 13 | 0.0429 | 0.1869 | 0.0085 | 0.0455 |
| LSC | psaB | 2205 | 2205 | 0 | - | 92 | 0.0417 | 0.1986 | 0.0041 | 0.0206 |
| LSC | psaA | 2253 | 2253 | 0 | - | 88 | 0.0391 | 0.1671 | 0.0064 | 0.0383 |
| LSC | ycf3 | 507 | 507 | 0 | - | 21 | 0.0414 | 0.2026 | 0.0050 | 0.0247 |
| LSC | rps4 | 606 | 606 | 0 | - | 37 | 0.0611 | 0.1864 | 0.0306 | 0.1642 |
| LSC | ndhJ | 477 | 477 | 0 | - | 26 | 0.0545 | 0.2322 | 0.0161 | 0.0693 |
| LSC | ndhK | 702 | 678 | 24 | 1 | 51 | 0.0752 | 0.2862 | 0.0273 | 0.0954 |
| LSC | ndhC | 363 | 363 | 0 | - | 14 | 0.0386 | 0.1533 | 0.0107 | 0.0698 |
| LSC | atpE | 402 | 423 | -21 | 1 | 34 | 0.0846 | 0.2346 | 0.0522 | 0.2225 |
| LSC | atpB | 1497 | 1497 | 0 | - | 88 | 0.0588 | 0.2387 | 0.0124 | 0.0519 |
| LSC | rbcL | 1434 | 1437 | -3 | 1 | 89 | 0.0621 | 0.1706 | 0.0358 | 0.2098 |
| LSC | accD | 1530 | 1515 | 15 | 4 | 149 | 0.0995 | 0.1790 | 0.0905 | 0.5056 |
| LSC | psaI | 111 | 111 | 0 | - | 11 | 0.0991 | 0.1953 | 0.0808 | 0.4137 |
| LSC | ycf4 | 549 | 555 | -6 | 1 | 47 | 0.0856 | 0.2304 | 0.0552 | 0.2396 |
| LSC | cemA | 690 | 690 | 0 | - | 45 | 0.0652 | 0.1773 | 0.0423 | 0.2386 |
| LSC | petA | 963 | 963 | 0 | - | 57 | 0.0592 | 0.1960 | 0.0260 | 0.1327 |
| LSC | psbJ | 123 | 123 | 0 | - | 6 | 0.0488 | 0.1752 | 0.0111 | 0.0634 |
| LSC | psbL | 117 | 117 | 0 | - | 2 | 0.0171 | 0.0903 | 0.0000 | 0.0000 |
| LSC | psbF | 120 | 120 | 0 | - | 5 | 0.0417 | 0.1450 | 0.0112 | 0.0772 |
| LSC | psbE | 252 | 252 | 0 | - | 9 | 0.0357 | 0.1363 | 0.0103 | 0.0756 |
| LSC | petL | 96 | 96 | 0 | - | 5 | 0.0521 | 0.1773 | 0.0143 | 0.0807 |
| LSC | petG | 114 | 114 | 0 | - | 0 | 0.0000 | 0.0000 | 0.0000 | - |
| LSC | psaJ | 135 | 129 | 6 | 1 | 7 | 0.0543 | 0.1773 | 0.0208 | 0.1173 |
| LSC | rpl33 | 201 | 201 | 0 | - | 18 | 0.0896 | 0.2512 | 0.0589 | 0.2345 |
| LSC | rps18 | 306 | 306 | 0 | - | 10 | 0.0327 | 0.0929 | 0.0170 | 0.1830 |
| LSC | rpl20 | 387 | 387 | 0 | - | 31 | 0.0801 | 0.2182 | 0.0500 | 0.2291 |
| LSC | clpP | 591 | 591 | 0 | - | 48 | 0.0812 | 0.1916 | 0.0571 | 0.2980 |
| LSC | psbB | 1527 | 1527 | 0 | - | 80 | 0.0524 | 0.2313 | 0.0086 | 0.0372 |
| LSC | psbT | 108 | 102 | 6 | 1 | 4 | 0.0392 | 0.1900 | 0.0000 | 0.0000 |
| LSC | psbN | 132 | 132 | 0 | - | 2 | 0.0152 | 0.0682 | 0.0000 | 0.0000 |
| LSC | psbH | 222 | 222 | 0 | - | 17 | 0.0766 | 0.1919 | 0.0489 | 0.2548 |
| LSC | petB | 648 | 648 | 0 | - | 32 | 0.0494 | 0.2389 | 0.0020 | 0.0084 |
| LSC | petD | 483 | 483 | 0 | - | 11 | 0.0228 | 0.1011 | 0.0000 | 0.0000 |
| LSC | rpoA | 1014 | 1062 | -48 | 3 | 79 | 0.0779 | 0.2021 | 0.0520 | 0.2573 |
| LSC | rps11 | 417 | 417 | 0 | - | 30 | 0.0719 | 0.2655 | 0.0196 | 0.0738 |
| LSC | rpl36 | 114 | 114 | 0 | - | 8 | 0.0702 | 0.3895 | 0.0000 | 0.0000 |
| LSC | infA | 234 | 234 | 0 | - | 14 | 0.0598 | 0.1946 | 0.0281 | 0.1444 |
| LSC | rps8 | 405 | 405 | 0 | - | 34 | 0.0840 | 0.2902 | 0.0379 | 0.1306 |
| LSC | rpl14 | 369 | 369 | 0 | - | 26 | 0.0705 | 0.2745 | 0.0216 | 0.0787 |
| LSC | rpl16 | 408 | 408 | 0 | - | 26 | 0.0637 | 0.2508 | 0.0163 | 0.0650 |
| LSC | rps3 | 663 | 660 | 3 | 1 | 44 | 0.0670 | 0.2742 | 0.0235 | 0.0857 |
| LSC | rpl22 | 468 | 483 | -15 | 3 | 68 | 0.1481 | 0.3589 | 0.1188 | 0.3310 |
| LSC | rps19 | 279 | 279 | 0 | - | 20 | 0.0717 | 0.2548 | 0.0306 | 0.1201 |
| LSC TOTAL | | 44127 | 44148 | -21 | 21 | 2711 | 0.0617 | 0.1511 | 0.0404 | 0.2674 |
| IR | rpl2 | 825 | 825 | 0 | - | 15 | 0.0182 | 0.0367 | 0.0128 | 0.3488 |
| IR | rpl23 | 285 | 282 | 3 | 1 | 3 | 0.0106 | 0.0000 | 0.0000 | - |
| IR | ycf2 | 6294 | 6333 | -39 | 20 | 162 | 0.0261 | 0.0396 | 0.0232 | 0.5859 |
| IR | ycf15 | 150 | 303 | -153 | 5 | 5 | 0.0347 | 0.0364 | 0.0278 | 0.7637 |
| IR | ndhB | 1533 | 1533 | 0 | - | 8 | 0.0052 | 0.0085 | 0.0043 | 0.5059 |
| IR | rps7 | 468 | 468 | 0 | - | 4 | 0.0085 | 0.0182 | 0.0056 | 0.3077 |
| IR | rrn16 | 1491 | 1491 | 0 | - | 5 | 0.0034 | - | - | - |
| IR | rrn23 | 2811 | 2809 | 2 | 2 | 30 | 0.0107 | - | - | - |
| IR | rrn4.5 | 103 | 103 | 0 | - | 1 | 0.0097 | - | - | - |
| IR | rrn5 | 121 | 121 | 0 | - | 0 | 0.0000 | - | - | - |
| IR TOTAL | | 14081 | 14268 | -187 | 17 | 233 | 0.0167 | 0.0296 | 0.0182 | 0.6149 |
| SSC | ndhF | 2256 | 2226 | 30 | 5 | 250 | 0.1128 | 0.3312 | 0.0725 | 0.2189 |
| SSC | rpl32 | 177 | 165 | 12 | 1 | 14 | 0.0848 | 0.2239 | 0.0578 | 0.2582 |
| SSC | ccsA | 978 | 963 | 15 | 2 | 110 | 0.1142 | 0.3244 | 0.0765 | 0.2358 |
| SSC | ndhD | 1503 | 1503 | 0 | - | 127 | 0.0845 | 0.2495 | 0.0477 | 0.1912 |
| SSC | psaC | 246 | 246 | 0 | - | 12 | 0.0488 | 0.2150 | 0.0105 | 0.0488 |
| SSC | ndhE | 306 | 306 | 0 | - | 20 | 0.0654 | 0.1624 | 0.0433 | 0.2666 |
| SSC | ndhG | 531 | 531 | 0 | - | 38 | 0.0716 | 0.2319 | 0.0328 | 0.1414 |
| SSC | ndhI | 507 | 504 | 3 | 1 | 38 | 0.0754 | 0.2347 | 0.0431 | 0.1836 |
| SSC | ndhA | 1092 | 1092 | 0 | - | 77 | 0.0705 | 0.1977 | 0.0389 | 0.1968 |
| SSC | ndhH | 1182 | 1182 | 0 | - | 96 | 0.0812 | 0.3345 | 0.0297 | 0.0888 |
| SSC | rps15 | 273 | 273 | 0 | - | 42 | 0.1538 | 0.4526 | 0.1092 | 0.2413 |
| SSC | ycf1 | 5370 | 5760 | -390 | 45 | 875 | 0.1683 | 0.2157 | 0.1860 | 0.8623 |
| SSC TOTAL | | 14421 | 14751 | -330 | 30 | 1699 | 0.1198 | 0.2447 | 0.1010 | 0.4128 |
| TOTAL | | 72629 | 73167 | -538 | 68 | 4643 | 0.0644 | 0.1502 | 0.0497 | 0.3309 |

Table S2. b) intergenic spacer region

| region | IGS | Size(Sesamum) | Size(Panax) | Indel | polymorphic site | nucleotide diversity | nucleotide diversity |
| --- | --- | --- | --- | --- | --- | --- | --- |
| LSC | tRNA-His/psbA | 292 | 404 | -112 | 65 | 0.2425 | 0.12994 |
| LSC | psbA/tRNA-Lys | 239 | 207 | 32 | 19 | 0.0927 | 0.09434 |
| LSC | tRNA-Lys/matK | 708 | 722 | -14 | 74 | 0.1076 | 0.04206 |
| LSC | matK/tRNA-Lys | 256 | 290 | -34 | 35 | 0.1434 | 0.12824 |
| LSC | tRNA-Lys/rps16 | 889 | 894 | -5 | 156 | 0.1940 | 0.18182 |
| LSC | rps16/tRnA-Gln | 1146 | 1796 | -650 | 217 | 0.2091 | 0.12901 |
| LSC | tRNA-Gln/psbK | 341 | 349 | -8 | 41 | 0.1209 | 0.08555 |
| LSC | psbK/psbI | 384 | 408 | -24 | 65 | 0.1733 | 0.11250 |
| LSC | psbI/tRNA-Ser | 120 | 138 | -18 | 18 | 0.1538 | 0.11594 |
| LSC | tRNA-Ser/tRNA-Gly | 703 | 727 | -24 | 113 | 0.1774 | 0.09897 |
| LSC | tRNA-Gly/tRNA-Arg | 188 | 168 | 20 | 52 | 0.3152 | 0.06977 |
| LSC | tRNA-Arg/atpA | 104 | 105 | -1 | 21 | 0.2188 | 0.14706 |
| LSC | atpA/atpF | 55 | 52 | 3 | 8 | 0.1538 | 0.03774 |
| LSC | atpF/atpH | 376 | 376 | 0 | 44 | 0.1222 | 0.11475 |
| LSC | atpH/atpI | 1001 | 1110 | -109 | 143 | 0.1529 | 0.10345 |
| LSC | atpI/rps2 | 230 | 241 | -11 | 34 | 0.1525 | 0.10638 |
| LSC | rps2/rpoC2 | 207 | 212 | -5 | 34 | 0.1700 | 0.15464 |
| LSC | rpoC2/rpoC1 | 154 | 165 | -11 | 19 | 0.1234 | 0.06173 |
| LSC | rpoC1/rpoB | 26 | 5 | 21 | 0 | 0.0000 | 0.00000 |
| LSC | rpoB/tRNA-Cys | 1163 | 1282 | -119 | 157 | 0.1408 | 0.13450 |
| LSC | tRNA-Cys/petN | 823 | 406 | 417 | 53 | 0.2062 | 0.11074 |
| LSC | petN/psbM | 981 | 862 | 119 | 105 | 0.1603 | 0.12795 |
| LSC | psbM/tRNA-Asp | 523 | 1145 | -622 | 74 | 0.1501 | 0.10779 |
| LSC | tRNA-Asp/tRNA-Tyr | 108 | 109 | -1 | 8 | 0.0748 | 0.08000 |
| LSC | tRNA-Tyr/tRNA-Glu | 59 | 59 | 0 | 3 | 0.0508 | 0.27869 |
| LSC | tRNA-Glu/tRNA-Thr | 549 | 807 | -258 | 87 | 0.1657 | 0.21280 |
| LSC | tRNA-Thr/psbD | 1325 | 1396 | -71 | 199 | 0.1563 | 0.12242 |
| LSC | psbD/psbC | - | 55 | - | - | - | 0.00000 |
| LSC | psbC/tRNA-Ser | 247 | 208 | 39 | 23 | 0.1106 | 0.06250 |
| LSC | tRNA-Ser/psbZ | 334 | 344 | -10 | 34 | 0.1093 | 0.13293 |
| LSC | psbZ/tRNA-Gly | 286 | 282 | 4 | 37 | 0.1386 | 0.11511 |
| LSC | tRNA-Gly/tRNA-fM | 177 | 178 | -1 | 35 | 0.2096 | 0.12209 |
| LSC | tRNA-fM/rps14 | 148 | 154 | -6 | 18 | 0.1216 | 0.06081 |
| LSC | rps14/psaB | 122 | 123 | -1 | 11 | 0.0902 | 0.05738 |
| LSC | psaB/psaA | 25 | 25 | 0 | 0 | 0.0000 | 0.00000 |
| LSC | psaA/ycf3 | 742 | 711 | 31 | 112 | 0.1647 | 0.08382 |
| LSC | ycf3/tRNA-Ser | 864 | 840 | 24 | 116 | 0.1491 | 0.11125 |
| LSC | tRNA-Ser/rps4 | 290 | 221 | 69 | 57 | 0.2664 | 0.12921 |
| LSC | rps4/tRNA-Thr | 351 | 344 | 7 | 57 | 0.1733 | 0.08438 |
| LSC | tRNA-Thr/tRNA-Leu | 694 | 796 | -102 | 114 | 0.1748 | 0.14305 |
| LSC | tRNA-Leu/tRNA-Phe | 341 | 361 | -20 | 66 | 0.1988 | 0.09146 |
| LSC | tRNA-Phe/ndhJ | 671 | 369 | 302 | 52 | 0.1465 | 0.10773 |
| LSC | ndhJ/ndhK | 75 | 105 | -30 | 13 | 0.1733 | 0.07692 |
| LSC | ndhK/ndhC | 53 | 48 | 5 | 4 | 0.0833 | 0.00000 |
| LSC | ndhC/tRNA-Val | 1156 | 1014 | 142 | 185 | 0.1939 | 0.14133 |
| LSC | tRNA-Val/tRNA-Met | 180 | 180 | 0 | 22 | 0.1264 | 0.09143 |
| LSC | tRNA-Met/atpB | 217 | 206 | 11 | 26 | 0.1376 | 0.19512 |
| LSC | atpB/Rbcl | 778 | 780 | -2 | 76 | 0.1008 | 0.09524 |
| LSC | Rbcl/accD | 635 | 643 | -8 | 80 | 0.1375 | 0.09344 |
| LSC | accD/psaI | 687 | 691 | -4 | 92 | 0.1438 | 0.14091 |
| LSC | psaI/ycf4 | 444 | 412 | 32 | 46 | 0.1159 | 0.07712 |
| LSC | ycf4/cemA | 876 | 325 | 551 | 0 | 0.0000 | 0.09231 |
| LSC | cemA/petA | 214 | 202 | 12 | 22 | 0.1164 | 0.11739 |
| LSC | petA/psbJ | 1004 | 1006 | -2 | 165 | 0.1750 | 0.11012 |
| LSC | psbJ/psbL | 133 | 132 | 1 | 6 | 0.0465 | 0.03788 |
| LSC | psbL/psbF | 23 | 22 | 1 | 0 | 0.0000 | 0.00000 |
| LSC | psbF/psbE | 14 | 9 | 5 | 1 | 0.1111 | 0.00000 |
| LSC | psbE/petL | 913 | 1173 | -260 | 129 | 0.1432 | 0.08723 |
| LSC | petL/petG | 181 | 152 | 29 | 25 | 0.1656 | 0.09868 |
| LSC | petG/tRNA-Trp | 127 | 121 | 6 | 29 | 0.2397 | 0.10744 |
| LSC | tRNA-Trp/tRNA-Pro | 163 | 134 | 29 | 18 | 0.1353 | 0.11024 |
| LSC | tRNA-Pro/psaJ | 389 | 389 | 0 | 44 | 0.1229 | 0.09511 |
| LSC | psaJ/rpl33 | 475 | 448 | 27 | 68 | 0.1655 | 0.11275 |
| LSC | rpl33/rps18 | 171 | 166 | 5 | 37 | 0.2387 | 0.06211 |
| LSC | rps18/rpl20 | 234 | 242 | -8 | 33 | 0.1410 | 0.15423 |
| LSC | rpl20/rps12-2 | 797 | 781 | 16 | 93 | 0.1214 | 0.06283 |
| LSC | rps12-2/clpP | 536 | 158 | 378 | 20 | 0.1504 | 0.15190 |
| LSC | clpP/psbB | 419 | 432 | -13 | 33 | 0.0838 | 0.08645 |
| LSC | psbB/psbT | 180 | 212 | -32 | 33 | 0.1844 | 0.11558 |
| LSC | psbT/psbN | 60 | 85 | -25 | 5 | 0.1136 | 0.02273 |
| LSC | psbN/psbH | 105 | 102 | 3 | 4 | 0.0392 | 0.02941 |
| LSC | psbH/petB | 124 | 131 | -7 | 19 | 0.1532 | 0.09091 |
| LSC | petB/petD | 188 | 174 | 14 | 20 | 0.1149 | 0.07429 |
| LSC | petD/rpoA | 181 | 115 | 66 | 10 | 0.1010 | 0.13559 |
| LSC | rpoA/rps11 | 71 | 74 | -3 | 10 | 0.1408 | 0.16418 |
| LSC | rps11/rpl36 | 101 | 19 | 82 | 17 | 0.1683 | 0.06364 |
| LSC | rpl36/infA | 95 | 115 | -20 | 12 | 0.1263 | 0.10435 |
| LSC | infA/rps8 | 124 | 117 | 7 | 14 | 0.1186 | 0.06612 |
| LSC | rps8/rpl14 | 180 | 191 | -11 | 28 | 0.1600 | 0.12707 |
| LSC | rpl14/rpl16 | 133 | 138 | -5 | 19 | 0.1545 | 0.18085 |
| LSC | rpl16/rps3 | 152 | 152 | 0 | 22 | 0.1618 | 0.14789 |
| LSC | rpl22/rps19 | 64 | 73 | -9 | 18 | 0.2857 | 0.17188 |
| LSC TOTAL |  | 30894 | 31115 | -221 | 4074 | 0.1494 | 0.11318 |

Table S2. b) (continued)

| region | IGS | Size(Sesamum) | Size(Panax) | Indel | number of INDEL events | number of polymorphic site | nucleotide diversity |
| --- | --- | --- | --- | --- | --- | --- | --- |
| IR | rps19/rpl2 | 64 | 53 | 11 | 4 | 0.0755 | 0.03774 |
| IR | rpl2/rpl23 | 18 | 18 | 0 | 0 | 0.0000 | 0.00000 |
| IR | rpl23/tRNA-His | 165 | 170 | -5 | 5 | 0.0303 | 0.01818 |
| IR | tRNA-His/ycf2 | 88 | 88 | 0 | 5 | 0.0568 | 0.00000 |
| IR | ycf2/ycf15 | 189 | 42 | 147 | 1 | 0.0238 | 0.07143 |
| IR | ycf15/tRNA-Leu | 359 | 320 | 39 | 14 | 0.0453 | 0.07317 |
| IR | tRNA-Leu/ndhB | 547 | 578 | -31 | 18 | 0.0333 | 0.02094 |
| IR | ndhB/rps7 | 274 | 297 | -23 | 5 | 0.0183 | 0.01347 |
| IR | rps7/rps12-2 | 53 | 53 | 0 | 1 | 0.0189 | 0.05660 |
| IR | rps12-2/tRNA-Val | 1603 | 1830 | -227 | 69 | 0.0441 | 0.08278 |
| IR | tRNA-Val/rrn16 | 227 | 227 | 0 | 6 | 0.0264 | 0.02203 |
| IR | rrn16/tRNA-Ile | 299 | 293 | 6 | 12 | 0.0410 | 0.03072 |
| IR | tRNA-Ile/tRNA-Ala | 64 | 64 | 0 | 2 | 0.0313 | 0.01563 |
| IR | tRNA-Ala/rrn23 | 157 | 152 | 5 | 4 | 0.0263 | 0.00769 |
| IR | rrn23/rrn4_5 | 98 | 98 | 0 | 1 | 0.0102 | 0.03061 |
| IR | rrn4_5/rrn5 | 255 | 242 | 13 | 6 | 0.0249 | 0.05357 |
| IR | rrn5/tRNA-Arg | 243 | 260 | -17 | 11 | 0.0453 | 0.01953 |
| IR | tRNA-Arg/tRNA-Asn | 568 | 588 | -20 | 40 | 0.0718 | 0.03691 |
| IR | tRNA-Asn/ycf1 | 328 | 327 | 1 | 8 | 0.0245 | 0.03067 |
| IR TOTAL |  | 5599 | 5700 | -101 | 212 | 0.0399 | 0.04707 |
| SSC | ndhF/rpl32 | 517 | 1218 | -701 | 84 | 0.1743 | 0.13810 |
| SSC | rpl32/tRNA-Leu | 883 | 814 | 69 | 166 | 0.2222 | 0.17488 |
| SSC | tRNA-Leu/ccsA | 91 | 80 | 11 | 18 | 0.2338 | 0.12346 |
| SSC | ccsA/ndhD | 252 | 220 | 32 | 36 | 0.1800 | 0.14354 |
| SSC | ndhD/psaC | 124 | 118 | 6 | 11 | 0.0932 | 0.02703 |
| SSC | psaC/ndhE | 250 | 248 | 2 | 34 | 0.1399 | 0.12903 |
| SSC | ndhE/ndhG | 225 | 218 | 7 | 40 | 0.2094 | 0.14747 |
| SSC | ndhG/ndhI | 347 | 378 | -31 | 77 | 0.2391 | 0.10588 |
| SSC | ndhI/ndhA | 79 | 82 | -3 | 9 | 0.1139 | 0.08434 |
| SSC | ndhA/ndhH | 1 | 1 | 0 | 0 | 0.0000 | 0.00000 |
| SSC | ndhH/rps15 | 97 | 92 | 5 | 14 | 0.1522 | 0.06593 |
| SSC | rps15/ycf1 | 367 | 391 | -24 | 82 | 0.2391 | 0.18852 |
| SSC TOTAL |  | 3233 | 3860 | -627 | 571 | 0.2082 | 0.14012 |
| TOTAL |  | 39726 | 40675 | -949 | 4857 | 0.1379 | 0.10561 |

*Table S2. c) intron region*

| region | intron | Size(Sesamum) | Size(Panax) | Indel | number of INDEL events | number of polymorphic site | nucleotide diversity |
| --- | --- | --- | --- | --- | --- | --- | --- |
| LSC | rps16 | 859 | 894 | -35 | 82 | 0.0989 | 0.10199 |
| LSC | tRNA-Gly | 688 | 697 | -9 | 75 | 0.1092 | 0.07914 |
| LSC | atpF | 695 | 730 | -35 | 73 | 0.1072 | 0.06331 |
| LSC | rpoC1 | 783 | 756 | 27 | 69 | 0.0950 | 0.06784 |
| LSC | ycf3 | 725 | 758 | -33 | 72 | 0.1043 | 0.06183 |
| LSC | ycf3 | 704 | 716 | -12 | 51 | 0.0735 | 0.07660 |
| LSC | tRNA-Leu | 488 | 507 | -19 | 32 | 0.0668 | 0.04208 |
| LSC | tRNA-Val | 578 | 578 | 0 | 43 | 0.0762 | 0.06572 |
| LSC | clpP | 630 | 632 | -2 | 83 | 0.1379 | 0.08475 |
| LSC | clpP | 739 | 771 | -32 | 67 | 0.0972 | 0.07712 |
| LSC | petB | 721 | 783 | -62 | 66 | 0.0932 | 0.06199 |
| LSC | petD | 733 | 751 | -18 | 79 | 0.1114 | 0.06649 |
| LSC | rpl16 | 886 | 944 | -58 | 130 | 0.1535 | 0.10337 |
| LSC total |  | 9229 | 9517 | -288 | 922 | 0.1035 | 0.07486 |
| IR | rpl2 | 667 | 660 | 7 | 8 | 0.0121 | 0.01846 |
| IR | ndhB | 679 | 678 | 1 | 14 | 0.0206 | 0.01917 |
| IR | rps12-2 | 536 | 536 | 0 | 3 | 0.0056 | 0.01679 |
| IR | tRNA-Ile | 950 | 945 | 5 | 12 | 0.0127 | 0.00745 |
| IR | tRNA-Ala | 812 | 808 | 4 | 17 | 0.0211 | 0.01115 |
| IR total |  | 3644 | 3627 | 17 | 54 | 0.0149 | 0.01385 |
| SSC | ndhA | 1080 | 1023 | 57 | 128 | 0.1292 | 0.10192 |
| SSC total |  | 1080 | 1023 | 57 | 128 | 0.1292 | 0.10192 |
| TOTAL |  | 13953 | 14167 | -214 | 1104 | 0.0817 | 0.06102 |
