# Supplementary material for: Complete Chloroplast Genome Sequences of Important Oilseed Crop Sesamum indicum L
Source: PLoS One. 2012 May 14;7(5):e35872. doi: 10.1371/journal.pone.0035872 (PMC3351433; doi:10.1371/journal.pone.0035872)
Supplement: Table S3 — Base substitutions and indels between Sesamum and Nicotiana; a) protein coding genes, b) intergenic spacer region and c) intron region. (DOC) [file pone.0035872.s003.doc]

Table S3. Base substitutions and indels between *Sesamum* and *Nicotiana.*

a) protein coding genes

| Region | Genes | Size(Sesamum) | Size(Nicotiana) | INDEL | number of INDEL events | Number of polymorphic site | Nucleotide diversity | Synonymous(Ks) | Nonsynonymous(Ka) | Ka/Ks |
| --- | --- | --- | --- | --- | --- | --- | --- | --- | --- | --- |
| LSC & IR | rps12 | 372 | 372 | 0 | - | 9 | 0.0244 | 0.0557 | 0.0146 | 0.2621 |
| LSC | psbA | 1059 | 1062 | -3 | 1 | 29 | 0.0275 | 0.1314 | 0.0000 | - |
| LSC | matK | 1530 | 1536 | -6 | 4 | 168 | 0.1113 | 0.2053 | 0.0990 | 0.4822 |
| LSC | rps16 | 255 | 258 | -3 | 2 | 20 | 0.0791 | 0.2349 | 0.0428 | 0.1822 |
| LSC | psbK | 186 | 186 | 0 | - | 16 | 0.0874 | 0.1367 | 0.0812 | 0.5940 |
| LSC | psbI | 111 | 111 | 0 | - | 6 | 0.0556 | 0.2737 | 0.0000 | - |
| LSC | atpA | 1524 | 1524 | 0 | - | 80 | 0.0526 | 0.2073 | 0.0130 | 0.0627 |
| LSC | atpF | 555 | 555 | 0 | - | 28 | 0.0507 | 0.1661 | 0.0236 | 0.1421 |
| LSC | atpH | 246 | 246 | 0 | - | 8 | 0.0329 | 0.1315 | 0.0000 | - |
| LSC | atpI | 744 | 744 | 0 | - | 32 | 0.0432 | 0.1867 | 0.0053 | 0.0284 |
| LSC | rps2 | 711 | 711 | 0 | - | 43 | 0.0607 | 0.2491 | 0.0183 | 0.0735 |
| LSC | rpoC2 | 4173 | 4152 | 21 | 7 | 308 | 0.0745 | 0.1408 | 0.0626 | 0.4446 |
| LSC | rpoC1 | 2055 | 2034 | 21 | 3 | 87 | 0.0429 | 0.1598 | 0.0141 | 0.0882 |
| LSC | rpoB | 3213 | 3213 | 0 | - | 178 | 0.0555 | 0.2123 | 0.0179 | 0.0843 |
| LSC | petN | 90 | 90 | 0 | - | 3 | 0.0345 | 0.1613 | 0.0000 | - |
| LSC | psbM | 105 | 105 | 0 | - | 4 | 0.0392 | 0.1800 | 0.0000 | - |
| LSC | psbD | 1062 | 1062 | 0 | - | 34 | 0.0321 | 0.1454 | 0.0025 | 0.0172 |
| LSC | psbC | 1422 | 1422 | 0 | - | 58 | 0.0409 | 0.1881 | 0.0019 | 0.0101 |
| LSC | psbZ | 189 | 189 | 0 | - | 6 | 0.0323 | 0.0914 | 0.0145 | 0.1586 |
| LSC | rps14 | 303 | 303 | 0 | - | 9 | 0.0300 | 0.1153 | 0.0086 | 0.0746 |
| LSC | psaB | 2205 | 2205 | 0 | - | 88 | 0.0400 | 0.1933 | 0.0029 | 0.0150 |
| LSC | psaA | 2253 | 2253 | 0 | - | 80 | 0.0356 | 0.1549 | 0.0046 | 0.0297 |
| LSC | ycf3 | 507 | 507 | 0 | - | 19 | 0.0377 | 0.1621 | 0.0089 | 0.0549 |
| LSC | rps4 | 606 | 606 | 0 | - | 27 | 0.0448 | 0.1255 | 0.0241 | 0.1920 |
| LSC | ndhJ | 477 | 477 | 0 | - | 19 | 0.0401 | 0.1820 | 0.0080 | 0.0440 |
| LSC | ndhK | 702 | 855 | -153 | 2 | 42 | 0.0622 | 0.2067 | 0.0274 | 0.1326 |
| LSC | ndhC | 363 | 363 | 0 | - | 21 | 0.0583 | 0.1611 | 0.0346 | 0.2148 |
| LSC | atpE | 402 | 423 | -21 | 1 | 32 | 0.0802 | 0.1881 | 0.0560 | 0.2977 |
| LSC | atpB | 1497 | 1497 | 0 | - | 77 | 0.0515 | 0.2014 | 0.0116 | 0.0576 |
| LSC | rbcL | 1434 | 1434 | 0 | - | 88 | 0.0615 | 0.1863 | 0.0311 | 0.1669 |
| LSC | accD | 1530 | 1539 | -9 | 4 | 148 | 0.0971 | 0.2086 | 0.0795 | 0.3811 |
| LSC | psaI | 111 | 111 | 0 | - | 8 | 0.0741 | 0.2273 | 0.0373 | 0.1641 |
| LSC | ycf4 | 549 | 555 | -6 | 2 | 43 | 0.0788 | 0.2158 | 0.0492 | 0.2280 |
| LSC | cemA | 690 | 690 | 0 | - | 51 | 0.0742 | 0.2172 | 0.0462 | 0.2127 |
| LSC | petA | 963 | 963 | 0 | - | 53 | 0.0552 | 0.1911 | 0.0219 | 0.1146 |
| LSC | psbJ | 123 | 123 | 0 | - | 5 | 0.0417 | 0.1391 | 0.0114 | 0.0820 |
| LSC | psbL | 117 | 117 | 0 | - | 1 | 0.0088 | 0.0000 | 0.0000 | - |
| LSC | psbF | 120 | 120 | 0 | - | 2 | 0.0171 | 0.0715 | 0.0000 | - |
| LSC | psbE | 252 | 252 | 0 | - | 8 | 0.0321 | 0.1585 | 0.0000 | - |
| LSC | petL | 96 | 96 | 0 | - | 3 | 0.0323 | 0.0858 | 0.0148 | 0.1725 |
| LSC | petG | 114 | 114 | 0 | - | 1 | 0.0090 | 0.0385 | 0.0000 | - |
| LSC | psaJ | 135 | 135 | 0 | - | 2 | 0.0152 | 0.0639 | 0.0000 | - |
| LSC | rpl33 | 201 | 201 | 0 | - | 14 | 0.0707 | 0.1319 | 0.0598 | 0.4534 |
| LSC | rps18 | 306 | 306 | 0 | - | 0 | 0.0000 | 0.0000 | 0.0000 | - |
| LSC | rpl20 | 387 | 387 | 0 | - | 28 | 0.0729 | 0.1835 | 0.0485 | 0.2643 |
| LSC | clpP | 591 | 591 | 0 | - | 37 | 0.0629 | 0.1970 | 0.0305 | 0.1548 |
| LSC | psbB | 1527 | 1527 | 0 | - | 66 | 0.0433 | 0.2019 | 0.0034 | 0.0168 |
| LSC | psbT | 108 | 108 | 0 | - | 2 | 0.0196 | 0.0435 | 0.0000 | - |
| LSC | psbN | 132 | 132 | 0 | - | 4 | 0.0310 | 0.1450 | 0.0000 | - |
| LSC | psbH | 222 | 222 | 0 | - | 15 | 0.0685 | 0.2439 | 0.0245 | 0.1005 |
| LSC | petB | 648 | 648 | 0 | - | 32 | 0.0496 | 0.2233 | 0.0061 | 0.0273 |
| LSC | petD | 483 | 483 | 0 | - | 14 | 0.0292 | 0.1324 | 0.0000 | - |
| LSC | rpoA | 1014 | 1062 | -48 | 1 | 66 | 0.0665 | 0.2005 | 0.0382 | 0.1905 |
| LSC | rps11 | 417 | 417 | 0 | - | 27 | 0.0652 | 0.2142 | 0.0232 | 0.1083 |
| LSC | rpl36 | 114 | 114 | 0 | - | 3 | 0.0270 | 0.1262 | 0.0000 | - |
| LSC | infA | 234 | 318 | -84 | 3 | 28 | 0.1279 | 0.2282 | 0.1178 | 0.5162 |
| LSC | rps8 | 405 | 405 | 0 | - | 31 | 0.0771 | 0.2968 | 0.0264 | 0.0889 |
| LSC | rpl14 | 369 | 372 | -3 | 1 | 14 | 0.0383 | 0.0997 | 0.0217 | 0.2177 |
| LSC | rpl16 | 408 | 408 | 0 | - | 22 | 0.0547 | 0.1988 | 0.0166 | 0.0835 |
| LSC | rps3 | 663 | 657 | 6 | 1 | 48 | 0.0734 | 0.2878 | 0.0288 | 0.1001 |
| LSC | rpl22 | 468 | 486 | -18 | 4 | 54 | 0.1184 | 0.2448 | 0.1009 | 0.4122 |
| LSC | rps19 | 279 | 279 | 0 | - | 22 | 0.0789 | 0.3425 | 0.0234 | 0.0683 |
| LSC TOTAL | | 44127 | 44433 | -306 | 22 | 2471 | 0.0564 | 0.1686 | 0.0280 | 0.1661 |
| IR | rpl2 | 825 | 825 | 0 | - | 7 | 0.0085 | 0.0156 | 0.0064 | 0.4103 |
| IR | rpl23 | 285 | 282 | 3 | 2 | 2 | 0.0072 | 0.0000 | 0.0000 | - |
| IR | ycf2 | 6294 | 6843 | -549 | 16 | 145 | 0.0234 | 0.0297 | 0.0219 | 0.7374 |
| IR | ycf15 | 150 | 264 | -114 | 3 | 4 | 0.0267 | 0.0349 | 0.0259 | 0.7421 |
| IR | ndhB | 1533 | 1533 | 0 | - | 17 | 0.0111 | 0.0377 | 0.0034 | 0.0902 |
| IR | rps7 | 468 | 468 | 0 | - | 6 | 0.0129 | 0.0183 | 0.0114 | 0.6230 |
| IR | rrn16 | 1491 | 1491 | 0 | - | 3 | 0.0020 | - | - | - |
| IR | rrn23 | 2811 | 2810 | 1 | 1 | 28 | 0.0100 | - | - | - |
| IR | rrn4.5 | 103 | 103 | 0 | - | 0 | 0.0000 | - | - | - |
| IR | rrn5 | 121 | 121 | 0 | - | 0 | 0.0000 | - | - | - |
| IR TOTAL | | 14081 | 14740 | -659 | 17 | 212 | 0.0152 | 0.0270 | 0.0170 | 0.6296 |
| SSC | ndhF | 2256 | 2241 | 15 | 4 | 252 | 0.1135 | 0.2984 | 0.0804 | 0.2694 |
| SSC | rpl32 | 177 | 168 | 9 | 2 | 19 | 0.1152 | 0.3924 | 0.0663 | 0.1690 |
| SSC | ccsA | 978 | 966 | 12 | 4 | 101 | 0.1076 | 0.3155 | 0.0680 | 0.2155 |
| SSC | ndhD | 1503 | 1503 | 0 | - | 128 | 0.0853 | 0.2505 | 0.0491 | 0.1960 |
| SSC | psaC | 246 | 246 | 0 | - | 13 | 0.0535 | 0.2959 | 0.0000 | 0.0000 |
| SSC | ndhE | 306 | 306 | 0 | - | 15 | 0.0495 | 0.1280 | 0.0304 | 0.2375 |
| SSC | ndhG | 531 | 531 | 0 | - | 39 | 0.0739 | 0.2297 | 0.0368 | 0.1602 |
| SSC | ndhI | 507 | 504 | 3 | 2 | 29 | 0.0579 | 0.2111 | 0.0248 | 0.1175 |
| SSC | ndhA | 1092 | 1092 | 0 | - | 77 | 0.0707 | 0.2101 | 0.0359 | 0.1709 |
| SSC | ndhH | 1182 | 1182 | 0 | - | 77 | 0.0653 | 0.2936 | 0.0169 | 0.0576 |
| SSC | rps15 | 273 | 273 | 0 | - | 28 | 0.1073 | 0.3118 | 0.0692 | 0.2219 |
| SSC | ycf1 | 5370 | 5802 | -432 | 3 | 34 | 0.0232 | 0.2773 | 0.1522 | 0.5489 |
| SSC TOTAL | | 14421 | 14814 | -393 | 30 | 1599 | 0.1125 | 0.2534 | 0.0878 | 0.3465 |
| TOTAL | | 72629 | 73987 | -1358 | 69 | 4282 | 0.0595 | 0.1630 | 0.0388 | 0.2380 |

Table S3. b) intergenic spacer region

| region | IGS | Size(Sesamum) | Size(Nicotiana) | Indel | polymorphic site | nucleotide diversity | nucleotide diversity |
| --- | --- | --- | --- | --- | --- | --- | --- |
| LSC | tRNA-His/psbA | 292 | 457 | -165 | 78 | 0.2847 | 0.26943 |
| LSC | psbA/tRNA-Lys | 239 | 214 | 25 | 30 | 0.1402 | 0.15942 |
| LSC | tRNA-Lys/matK | 708 | 712 | -4 | 75 | 0.1078 | 0.10480 |
| LSC | matK/tRNA-Lys | 256 | 284 | -28 | 84 | 0.3281 | 0.07052 |
| LSC | tRNA-Lys/rps16 | 889 | 687 | 202 | 123 | 0.2023 | 0.16323 |
| LSC | rps16/tRnA-Gln | 1146 | 1205 | -59 | 285 | 0.2775 | 0.19914 |
| LSC | tRNA-Gln/psbK | 341 | 347 | -6 | 36 | 0.1078 | 0.12275 |
| LSC | psbK/psbI | 384 | 377 | 7 | 57 | 0.1575 | 0.15761 |
| LSC | psbI/tRNA-Ser | 120 | 126 | -6 | 15 | 0.1351 | 0.19328 |
| LSC | tRNA-Ser/tRNA-Gly | 703 | 779 | -76 | 115 | 0.1742 | 0.17960 |
| LSC | tRNA-Gly/tRNA-Arg | 188 | 169 | 19 | 31 | 0.1950 | 0.17021 |
| LSC | tRNA-Arg/atpA | 104 | 123 | -19 | 13 | 0.1262 | 0.17647 |
| LSC | atpA/atpF | 55 | 54 | 1 | 6 | 0.1111 | 0.14815 |
| LSC | atpF/atpH | 376 | 401 | -25 | 37 | 0.1003 | 0.14846 |
| LSC | atpH/atpI | 1001 | 1157 | -156 | 125 | 0.1323 | 0.15706 |
| LSC | atpI/rps2 | 230 | 226 | 4 | 18 | 0.0845 | 0.12903 |
| LSC | rps2/rpoC2 | 207 | 227 | -20 | 24 | 0.1159 | 0.15459 |
| LSC | rpoC2/rpoC1 | 154 | 158 | -4 | 21 | 0.1438 | 0.13699 |
| LSC | rpoC1/rpoB | 26 | 5 | 21 | 0 | 0.0000 | 0.00000 |
| LSC | rpoB/tRNA-Cys | 1163 | 1284 | -121 | 146 | 0.1307 | 0.16428 |
| LSC | tRNA-Cys/petN | 823 | 670 | 153 | 78 | 0.1324 | 0.15101 |
| LSC | petN/psbM | 981 | 1132 | -151 | 138 | 0.1497 | 0.16974 |
| LSC | psbM/tRNA-Asp | 523 | 1070 | -547 | 57 | 0.1145 | 0.18263 |
| LSC | tRNA-Asp/tRNA-Tyr | 108 | 108 | 0 | 11 | 0.1019 | 0.08411 |
| LSC | tRNA-Tyr/tRNA-Glu | 59 | 59 | 0 | 1 | 0.0169 | 0.06780 |
| LSC | tRNA-Glu/tRNA-Thr | 549 | 848 | -299 | 76 | 0.1462 | 0.18024 |
| LSC | tRNA-Thr/psbD | 1325 | 1217 | 108 | 156 | 0.1435 | 0.15004 |
| LSC | psbD/psbC | - | - | - | - | - | 0.00000 |
| LSC | psbC/tRNA-Ser | 247 | 239 | 8 | 39 | 0.1681 | 0.13000 |
| LSC | tRNA-Ser/psbZ | 334 | 362 | -28 | 30 | 0.0915 | 0.11818 |
| LSC | psbZ/tRNA-Gly | 286 | 275 | 11 | 38 | 0.1456 | 0.16981 |
| LSC | tRNA-Gly/tRNA-fM | 177 | 227 | -50 | 28 | 0.1697 | 0.17442 |
| LSC | tRNA-fM/rps14 | 148 | 149 | -1 | 17 | 0.1156 | 0.11486 |
| LSC | rps14/psaB | 122 | 122 | 0 | 13 | 0.1074 | 0.09836 |
| LSC | psaB/psaA | 25 | 25 | 0 | 1 | 0.0400 | 0.04000 |
| LSC | psaA/ycf3 | 742 | 752 | -10 | 88 | 0.1317 | 0.12412 |
| LSC | ycf3/tRNA-Ser | 864 | 854 | 10 | 122 | 0.1529 | 0.14925 |
| LSC | tRNA-Ser/rps4 | 290 | 338 | -48 | 48 | 0.1702 | 0.17361 |
| LSC | rps4/tRNA-Thr | 351 | 370 | -19 | 56 | 0.1652 | 0.13354 |
| LSC | tRNA-Thr/tRNA-Leu | 694 | 710 | -16 | 92 | 0.1465 | 0.16763 |
| LSC | tRNA-Leu/tRNA-Phe | 341 | 356 | -15 | 47 | 0.1424 | 0.18927 |
| LSC | tRNA-Phe/ndhJ | 671 | 676 | -5 | 98 | 0.1563 | 0.16898 |
| LSC | ndhJ/ndhK | 75 | 105 | -30 | 11 | 0.1467 | 0.12381 |
| LSC | ndhK/ndhC | 53 | 12 | 41 | 6 | 0.5000 | 0.50000 |
| LSC | ndhC/tRNA-Val | 1156 | 1086 | 70 | 168 | 0.1658 | 0.16970 |
| LSC | tRNA-Val/tRNA-Met | 180 | 190 | -10 | 17 | 0.0944 | 0.09827 |
| LSC | tRNA-Met/atpB | 217 | 221 | -4 | 27 | 0.1324 | 0.16129 |
| LSC | atpB/Rbcl | 778 | 817 | -39 | 61 | 0.0803 | 0.09855 |
| LSC | Rbcl/accD | 635 | 764 | -129 | 69 | 0.1154 | 0.13688 |
| LSC | accD/psaI | 687 | 751 | -64 | 76 | 0.1146 | 0.15648 |
| LSC | psaI/ycf4 | 444 | 446 | -2 | 49 | 0.1129 | 0.13317 |
| LSC | ycf4/cemA | 876 | 222 | 654 | 32 | 0.1509 | 0.15766 |
| LSC | cemA/petA | 214 | 230 | -16 | 24 | 0.1148 | 0.11013 |
| LSC | petA/psbJ | 1004 | 1065 | -61 | 179 | 0.1906 | 0.20555 |
| LSC | psbJ/psbL | 133 | 124 | 9 | 9 | 0.0732 | 0.08130 |
| LSC | psbL/psbF | 23 | 22 | 1 | 1 | 0.0455 | 0.04545 |
| LSC | psbF/psbE | 14 | 11 | 3 | 0 | 0.0000 | 0.11111 |
| LSC | psbE/petL | 913 | 1163 | -250 | 130 | 0.1471 | 0.12414 |
| LSC | petL/petG | 181 | 181 | 0 | 28 | 0.1609 | 0.11842 |
| LSC | petG/tRNA-Trp | 127 | 131 | -4 | 15 | 0.1210 | 0.14530 |
| LSC | tRNA-Trp/tRNA-Pro | 163 | 166 | -3 | 25 | 0.1534 | 0.12687 |
| LSC | tRNA-Pro/psaJ | 389 | 438 | -49 | 48 | 0.1244 | 0.13978 |
| LSC | psaJ/rpl33 | 475 | 431 | 44 | 56 | 0.1349 | 0.14805 |
| LSC | rpl33/rps18 | 171 | 186 | -15 | 31 | 0.1813 | 0.17073 |
| LSC | rps18/rpl20 | 234 | 199 | 35 | 15 | 0.0785 | 0.11856 |
| LSC | rpl20/rps12-2 | 797 | 809 | -12 | 72 | 0.0935 | 0.09987 |
| LSC | rps12-2/clpP | 536 | 138 | 398 | 16 | 0.1203 | 0.12030 |
| LSC | clpP/psbB | 419 | 445 | -26 | 41 | 0.0988 | 0.11557 |
| LSC | psbB/psbT | 180 | 200 | -20 | 36 | 0.2034 | 0.15152 |
| LSC | psbT/psbN | 60 | 73 | -13 | 9 | 0.1500 | 0.15909 |
| LSC | psbN/psbH | 105 | 111 | -6 | 9 | 0.0857 | 0.03922 |
| LSC | psbH/petB | 124 | 129 | -5 | 19 | 0.1532 | 0.17829 |
| LSC | petB/petD | 188 | 190 | -2 | 23 | 0.1257 | 0.14045 |
| LSC | petD/rpoA | 181 | 187 | -6 | 21 | 0.1280 | 0.12613 |
| LSC | rpoA/rps11 | 71 | 65 | 6 | 13 | 0.2000 | 0.20000 |
| LSC | rps11/rpl36 | 101 | 101 | 0 | 13 | 0.1287 | 0.14851 |
| LSC | rpl36/infA | 95 | 12 | 83 | 1 | 0.0833 | 0.16667 |
| LSC | infA/rps8 | 124 | 106 | 18 | 12 | 0.1132 | 0.11321 |
| LSC | rps8/rpl14 | 180 | 168 | 12 | 23 | 0.1420 | 0.16867 |
| LSC | rpl14/rpl16 | 133 | 124 | 9 | 19 | 0.1624 | 0.22222 |
| LSC | rpl16/rps3 | 152 | 146 | 6 | 17 | 0.1250 | 0.13287 |
| LSC | rpl22/rps19 | 64 | 53 | 11 | 8 | 0.1509 | 0.16981 |
| LSC TOTAL |  | 30894 | 31569 | -675 | 3982 | 0.1462 | 0.15079 |

Table S3. b) (continued)

| region | IGS | Size(Sesamum) | Size(Nicotiana) | Indel | number of INDEL events | number of polymorphic site | nucleotide diversity |
| --- | --- | --- | --- | --- | --- | --- | --- |
| IR | rps19/rpl2 | 64 | 60 | 4 | 5 | 0.0847 | 0.07547 |
| IR | rpl2/rpl23 | 18 | 18 | 0 | 2 | 0.1111 | 0.11111 |
| IR | rpl23/tRNA-His | 165 | 167 | -2 | 5 | 0.0303 | 0.00606 |
| IR | tRNA-His/ycf2 | 88 | 112 | -24 | 3 | 0.0341 | 0.02273 |
| IR | ycf2/ycf15 | 189 | 90 | 99 | 2 | 0.0222 | 0.00000 |
| IR | ycf15/tRNA-Leu | 359 | 311 | 48 | 8 | 0.0259 | 0.02903 |
| IR | tRNA-Leu/ndhB | 547 | 539 | 8 | 20 | 0.0382 | 0.03525 |
| IR | ndhB/rps7 | 274 | 279 | -5 | 5 | 0.0186 | 0.01799 |
| IR | rps7/rps12-2 | 53 | 53 | 0 | 1 | 0.0189 | 0.00000 |
| IR | rps12-2/tRNA-Val | 1603 | 1607 | -4 | 54 | 0.0348 | 0.04525 |
| IR | tRNA-Val/rrn16 | 227 | 227 | 0 | 6 | 0.0264 | 0.03084 |
| IR | rrn16/tRNA-Ile | 299 | 300 | -1 | 10 | 0.0334 | 0.02730 |
| IR | tRNA-Ile/tRNA-Ala | 64 | 64 | 0 | 0 | 0.0000 | 0.03125 |
| IR | tRNA-Ala/rrn23 | 157 | 153 | 4 | 5 | 0.0327 | 0.01316 |
| IR | rrn23/rrn4_5 | 98 | 101 | -3 | 1 | 0.0102 | 0.01020 |
| IR | rrn4_5/rrn5 | 255 | 256 | -1 | 3 | 0.0118 | 0.01653 |
| IR | rrn5/tRNA-Arg | 243 | 257 | -14 | 9 | 0.0370 | 0.04781 |
| IR | tRNA-Arg/tRNA-Asn | 568 | 581 | -13 | 32 | 0.0575 | 0.05789 |
| IR | tRNA-Asn/ycf1 | 328 | 325 | 3 | 9 | 0.0277 | 0.02469 |
| IR TOTAL |  | 5599 | 5500 | 99 | 180 | 0.0337 | 0.03557 |
| SSC | ndhF/rpl32 | 517 | 768 | -251 | 89 | 0.1850 | 0.21728 |
| SSC | rpl32/tRNA-Leu | 883 | 932 | -49 | 188 | 0.2333 | 0.24198 |
| SSC | tRNA-Leu/ccsA | 91 | 103 | -12 | 15 | 0.1648 | 0.20988 |
| SSC | ccsA/ndhD | 252 | 237 | 15 | 51 | 0.2406 | 0.23618 |
| SSC | ndhD/psaC | 124 | 118 | 6 | 17 | 0.1441 | 0.07759 |
| SSC | psaC/ndhE | 250 | 260 | -10 | 40 | 0.1660 | 0.15702 |
| SSC | ndhE/ndhG | 225 | 223 | 2 | 38 | 0.1979 | 0.16514 |
| SSC | ndhG/ndhI | 347 | 396 | -49 | 81 | 0.2455 | 0.21512 |
| SSC | ndhI/ndhA | 79 | 84 | -5 | 11 | 0.1392 | 0.13415 |
| SSC | ndhA/ndhH | 1 | 1 | 0 | 0 | 0.0000 | 0.00000 |
| SSC | ndhH/rps15 | 97 | 111 | -14 | 14 | 0.1443 | 0.08696 |
| SSC | rps15/ycf1 | 367 | 397 | -30 | 66 | 0.1919 | 0.23098 |
| SSC TOTAL |  | 3233 | 3630 | -397 | 610 | 0.2039 | 0.20645 |
| TOTAL |  | 39726 | 40699 | -973 | 4772 | 0.1343 | 0.13923 |

*Table S3. c) intron region*

| region | intron | Size(Sesamum) | Size(Nicotiana) | Indel | number of INDEL events | number of polymorphic site | nucleotide diversity |
| --- | --- | --- | --- | --- | --- | --- | --- |
| LSC | rps16 | 859 | 860 | -1 | 91 | 0.1102 | 0.12395 |
| LSC | tRNA-Gly | 688 | 692 | -4 | 64 | 0.0937 | 0.09503 |
| LSC | atpF | 695 | 695 | 0 | 63 | 0.0936 | 0.09738 |
| LSC | rpoC1 | 783 | 737 | 46 | 66 | 0.0922 | 0.11172 |
| LSC | ycf3 | 724 | 783 | -59 | 63 | 0.0889 | 0.09973 |
| LSC | ycf3 | 704 | 738 | -34 | 50 | 0.0715 | 0.07989 |
| LSC | tRNA-Leu | 488 | 503 | -15 | 29 | 0.0612 | 0.07010 |
| LSC | tRNA-Val | 578 | 571 | 7 | 39 | 0.0694 | 0.08021 |
| LSC | clpP | 630 | 637 | -7 | 63 | 0.1029 | 0.11909 |
| LSC | clpP | 739 | 807 | -68 | 67 | 0.0938 | 0.10719 |
| LSC | petB | 721 | 753 | -32 | 66 | 0.0973 | 0.08560 |
| LSC | petD | 733 | 742 | -9 | 75 | 0.1068 | 0.10833 |
| LSC | rpl16 | 886 | 1020 | -134 | 100 | 0.1175 | 0.12361 |
| LSC total |  | 9228 | 9538 | -310 | 836 | 0.0939 | 0.10185 |
| IR | rpl2 | 667 | 666 | 1 | 9 | 0.0135 | 0.01517 |
| IR | ndhB | 679 | 679 | 0 | 8 | 0.0118 | 0.02651 |
| IR | rps12-2 | 536 | 536 | 0 | 0 | 0.0000 | 0.00560 |
| IR | tRNA-Ile | 950 | 707 | 243 | 7 | 0.0099 | 0.00850 |
| IR | tRNA-Ala | 812 | 709 | 103 | 11 | 0.0155 | 0.01280 |
| IR total |  | 3644 | 3297 | 347 | 35 | 0.0106 | 0.01401 |
| SSC | ndhA | 1080 | 1148 | -68 | 141 | 0.1326 | 0.14776 |
| SSC total |  | 1080 | 1148 | -68 | 141 | 0.1326 | 0.14776 |
| TOTAL |  | 13952 | 13983 | -31 | 1012 | 0.0763 | 0.08398 |
