# Supplementary material for: Complete Chloroplast Genome Sequences of Important Oilseed Crop Sesamum indicum L
Source: PLoS One. 2012 May 14;7(5):e35872. doi: 10.1371/journal.pone.0035872 (PMC3351433; doi:10.1371/journal.pone.0035872)
Supplement: Table S4 — The distribution patterns of indel numbers and indel sizes. (DOC) [file pone.0035872.s004.doc]

Table S4. The distribution patterns of indel numbers and indel sizes.

| size of indel Number | 1 | 2 | 3 | 4 | 5 | 6 | 7 | 8 | 9 | 10 | 11 | 12 | 13 | 14 | 15 | 16 | 17 | 18 | 19 | 20 | 21 | 22 | 23 | 24 | 25 |
| --- | --- | --- | --- | --- | --- | --- | --- | --- | --- | --- | --- | --- | --- | --- | --- | --- | --- | --- | --- | --- | --- | --- | --- | --- | --- |
| Sesame vs. Olea | 218 | 91 | 56 | 53 | 71 | 86 | 29 | 29 | 56 | 24 | 20 | 7 | 14 | 4 | 5 | 2 | 3 | 7 | 3 | 0 | 5 | 0 | 3 | 2 | 1 |
| Sesame vs. Nicotiana | 228 | 105 | 153 | 86 | 128 | 112 | 59 | 52 | 75 | 33 | 19 | 20 | 17 | 14 | 15 | 6 | 8 | 7 | 2 | 9 | 10 | 4 | 3 | 2 | 3 |
| Sesame vs. Panax | 255 | 107 | 119 | 88 | 131 | 106 | 52 | 47 | 63 | 38 | 25 | 18 | 10 | 12 | 12 | 4 | 8 | 7 | 10 | 4 | 7 | 4 | 4 | 1 | 2 |
| size of indel Number | 26 | 27 | 28 | 29 | 30 | 31 | 32 | 33 | 34 | 35 | 36 | 37 | 38 | 39 | 40 | 41 | 42 | 43 | 44 | 45 | 46 | 47 | 48 | 49 | 50 |
| Sesame vs. Olea | 1 | 4 | 0 | 1 | 1 | 0 | 2 | 1 | 0 | 0 | 0 | 1 | 0 | 1 | 0 | 1 | 1 | 0 | 0 | 0 | 0 | 0 | 1 | 0 | 1 |
| Sesame vs. Nicotiana | 0 | 1 | 4 | 1 | 5 | 3 | 2 | 2 | 1 | 0 | 3 | 3 | 2 | 0 | 1 | 2 | 0 | 2 | 0 | 0 | 1 | 2 | 0 | 0 | 3 |
| Sesame vs. Panax | 0 | 2 | 2 | 2 | 3 | 1 | 1 | 2 | 4 | 3 | 2 | 2 | 2 | 1 | 1 | 2 | 1 | 0 | 0 | 0 | 1 | 0 | 2 | 0 | 1 |
