# Supplementary material for: Complete Chloroplast Genome Sequences of Important Oilseed Crop Sesamum indicum L
Source: PLoS One. 2012 May 14;7(5):e35872. doi: 10.1371/journal.pone.0035872 (PMC3351433; doi:10.1371/journal.pone.0035872)
Supplement: Table S5 — The list of accession number of the chloroplast genome sequences used in this study. (DOC) [file pone.0035872.s005.doc]

Table S5. The list of accession number of the chloroplast genome sequences used in this study.

| Taxon | Order | GenBank Accession Number |
| --- | --- | --- |
| *Ageratina adenophora* | Asterales | NC_015621 |
| *Anethum graveolens* | Apiales | EU016721-EU016801(incomplete) |
| *Anthriscus cerefolium* | Apiales | NC_015113 |
| *Arabidopsis thaliana* | Brassicales | NC_000932 |
| *Atropa belladonna* | Solanales | NC_004561 |
| *Coffea arabica* | Gentianales | NC_008535 |
| *Crithmum maritimum* | Apiales | NC_015804 |
| *Daucus carota* | Apiales | NC_008325 |
| *Guizotia abyssinica* | Asterales | NC_010601 |
| *Helianthus annuus* | Asterales | NC_007977 |
| *Hydrocotyle* sp. | Apiales | NC_015818 |
| *Ipomoea purpurea* | Solanales | NC_009808 |
| *Jacobaea vulgaris* | Asterales | NC_015543 |
| *Jasminum nudiflorum* | Lamiales | NC_008407 |
| *Lactuca sativa* | Asterales | NC_007578 |
| *Nicotiana sylvestris* | Solanales | NC_007500 |
| *Nicotiana tabacum* | Solanales | NC_001879 |
| *Nicotiana tomentosiformis* | Solanales | NC_007602 |
| *Olea europaea* *cv.* Bianchera | Lamiales | NC_013707 |
| *Olea europaea* subsp. *cuspidata* | Lamiales | NC_015604 |
| *Olea europaea* subsp. *europaea* *cv.* Manzanilla | Lamiales | FN996972 |
| *Olea europaea* subsp. *maroccana* | Lamiales | NC_015623 |
| *Olea europaea* subsp. *woodiana* | Lamiales | NC_015608 |
| *Oxypolis greenmanii* | Apiales | NC_015832 |
| *Panax ginseng* | Apiales | NC_006290 |
| *Petroselinum crispum* | Apiales | NC_015821 |
| *Scaevola aemula* | Asterales | EU017139-EU017217(incomplete) |
| *Solanum bulbocastanum* | Solanales | NC_007943 |
| *Solanum lycopersicum* | Solanales | NC_007898 |
| *Solanum tuberosum* | Solanales | NC_008096 |
| *Spinacia oleracea* | Caryophyllales | NC_002202 |
| *Trachelium caeruleum* | Asterales | NC_010442 |
